# A glycoprotein D-targeted lipid nanoparticle-encapsulated mRNA vaccine elicits strong protective immunity against pseudorabies virus

**DOI:** 10.1128/jvi.01472-25

**Published:** 2025-11-06

**Authors:** Yue Sun, Shi-Jia Xu, Yongfei Zhou, Yanhe Zhang, Hongliang Zhang, Ting Le, Yuan-Zhe Bai, Cui-Hong Rao, Shanshan Huo, Tianceng Zhou, Tong-Qing An, Xin Yin, Fei Yu, Xue-Hui Cai, Yan-Dong Tang

**Affiliations:** 1 State Key Laboratory for Animal Disease Control and Prevention, Harbin Veterinary Research Institute of Chinese Academy of Agricultural Sciences 111613 , Harbin, China; 2 Hebei Key Laboratory of Analysis and Control of Zoonotic Pathogenic Microorganism, College of Life Sciences, Hebei Agricultural University 162640 , Baoding, China; 3 College of Animal Science and Veterinary Medicine, Heilongjiang Bayi Agricultural University, Daqing, China; 4 HBAU-Hemu Joint R&D Center for Animal mRNA Vaccine, Daqing, China; 5 Heilongjiang Provincial Research Center for Veterinary Biomedicine, Harbin Veterinary Research Institute of Chinese Academy of Agricultural Sciences, Harbin, China; 6 Heilongjiang Provincial Key Laboratory of Veterinary Immunology, Harbin Veterinary Research Institute of Chinese Academy of Agricultural Sciences, Harbin, China; University of Toronto, Toronto, Ontario, Canada

**Keywords:** mRNA vaccine, pseudorabies virus, glycoprotein D, vaccine

## Abstract

The pseudorabies virus (PRV), a highly contagious pathogen with zoonotic potential, continues to threaten swine production and public health due to the emergence of virulent variants and insufficient protection conferred by conventional live attenuated vaccines. Although commercial vaccines are safe for pigs, their residual pathogenicity in other susceptible species underscores the demand for universally safe alternatives. Here, we engineered a lipid nanoparticle-encapsulated mRNA vaccine (mRNA-LNPs) expressing PRV glycoprotein D (gD) and evaluated its efficacy in murine and porcine models. In mice, vaccination with gD mRNA-LNPs elicited potent neutralizing antibodies and provided complete protection against lethal PRV challenge. In piglets, immunization induced rapid humoral immune responses, significantly reduced viral loads in tissues and viral shedding post-challenge, and alleviated histopathological damage. Mechanistically, except for its ability to elicit neutralizing antibodies, the vaccine also stimulated antigen-specific CD3^+^CD4^+^ T-cell and CD3^+^CD8^+^ T-cell proliferation and enhanced IFN-γ production, demonstrating robust activation of both humoral and cellular immunity. These findings establish gD mRNA-LNPs as a safe, effective, and broadly applicable vaccine candidate for PRV control across susceptible species, with advantages in scalability and biosafety over traditional platforms.

**IMPORTANCE:**

The emergence of virulent pseudorabies virus (PRV) variants and the insufficient cross-species protection conferred by conventional live attenuated vaccines pose significant challenges to global swine production and zoonotic biosecurity. Here, we developed a lipid nanoparticle-encapsulated mRNA vaccine (gD mRNA-LNPs) targeting PRV glycoprotein D (gD), a critical mediator of viral entry. This vaccine elicits robust neutralizing antibodies and potent T-cell responses, providing complete protection against lethal PRV challenge in both murine and porcine models. Unlike traditional vaccines, gD mRNA-LNPs eliminates residual pathogenicity risks and demonstrates broad efficacy against diverse PRV strains, including emerging variants. Its scalable production platform and ability to differentiate vaccinated from infected animals via serological diagnostics align with One Health strategies for PRV eradication. This study establishes mRNA-LNPs technology as a versatile, safe, and effective solution for combating PRV, with implications for improving livestock health and reducing zoonotic spillover threats.

## INTRODUCTION

Pseudorabies virus (PRV), a member of the *Alphaherpesvirinae* subfamily (Herpesviridae), is a highly virulent pathogen that imposes substantial burdens on global swine industries and zoonotic biosecurity (1). As the primary natural host, pigs exhibit a spectrum of clinical manifestations upon PRV infection, ranging from respiratory distress and neurological dysfunction to reproductive failure and mortality, with severity modulated by age, immunological competence, and viral genotype (2, 3). While pigs serve as the natural reservoir, PRV demonstrates alarming cross-species tropism, infecting ruminants, carnivores, and rodents with near-uniform lethality, thereby amplifying its ecological threat (4–6). The emergence of genotype II variants in China since 2011 has exacerbated economic losses in pig farming and heightened spillover risks, including sporadic human infections, underscoring the urgency for next-generation prophylactic strategies (7, 8). The most widely utilized commercial PRV vaccines are based on the Bartha K61 strain, which was developed through the attenuation of virulent strains via serial passaging on chicken embryo fibroblasts, similar to other traditional live attenuated vaccines (9–11). The prevention and control of PRV in China and worldwide mainly rely on the administration of the live attenuated vaccine Bartha K61 (2, 3, 10). However, since 2011, PRV variants have emerged in China, rendering the efficacy of the Bartha K61 vaccine inadequate in providing sufficient immune protection (6). Moreover, different animal species exhibit varying degrees of susceptibility to PRV, with some animals still experiencing high pathogenicity toward live attenuated vaccine (12). Therefore, it is imperative to develop a universal vaccine that ensures safety and effectiveness across all susceptible animal hosts for combating this inter-species transmission.

mRNA vaccine technology has emerged as a transformative platform for combating viral pathogens, offering unparalleled advantages in safety, scalability, and rapid adaptability (13). mRNA vaccines can be classified into two types: self-amplifying RNA (saRNA) and non-replicating mRNA. The conventional non-replicating mRNA consists of a cap, 5′-untranslated regions (UTR), open reading frame (ORF) encoding protective antigens, 3′-UTRs, and poly(A) tail. Apart from the ORF, these structural elements play a crucial role in maintaining mRNA stability and transcriptional efficiency. Moreover, they can be modified to extend the half-life of mRNA *in vivo* and minimize undesired immune responses (13, 14). The success of mRNA vaccines against SARS-CoV-2 (15, 16), influenza (17), Zika virus (18), and porcine coronaviruses (e.g., PEDV, PDCoV) underscores their versatility and positions them as a paradigm-shifting solution for addressing various viral challenges (19, 20).

Here, we present a lipid nanoparticle-encapsulated mRNA vaccine encoding the glycoprotein D (gD) of PRV (gD mRNA-LNPs), engineered to address the limitations of existing vaccines. By leveraging gD as the immunodominant antigen, this candidate induces potent neutralizing antibodies and antigen-specific T-cell responses, conferring cross-species protection against high-dose PRV challenge in mice and piglets. This research not only redefines the role of gD in PRV immunoprotection but also establishes a blueprint for mRNA-based interventions against alphaherpesviruses, with implications for mitigating zoonotic transmission and advancing One Health initiatives.

## RESULTS

### Design and *in vitro* validation of gD mRNA-LNP

**Fig 1:**
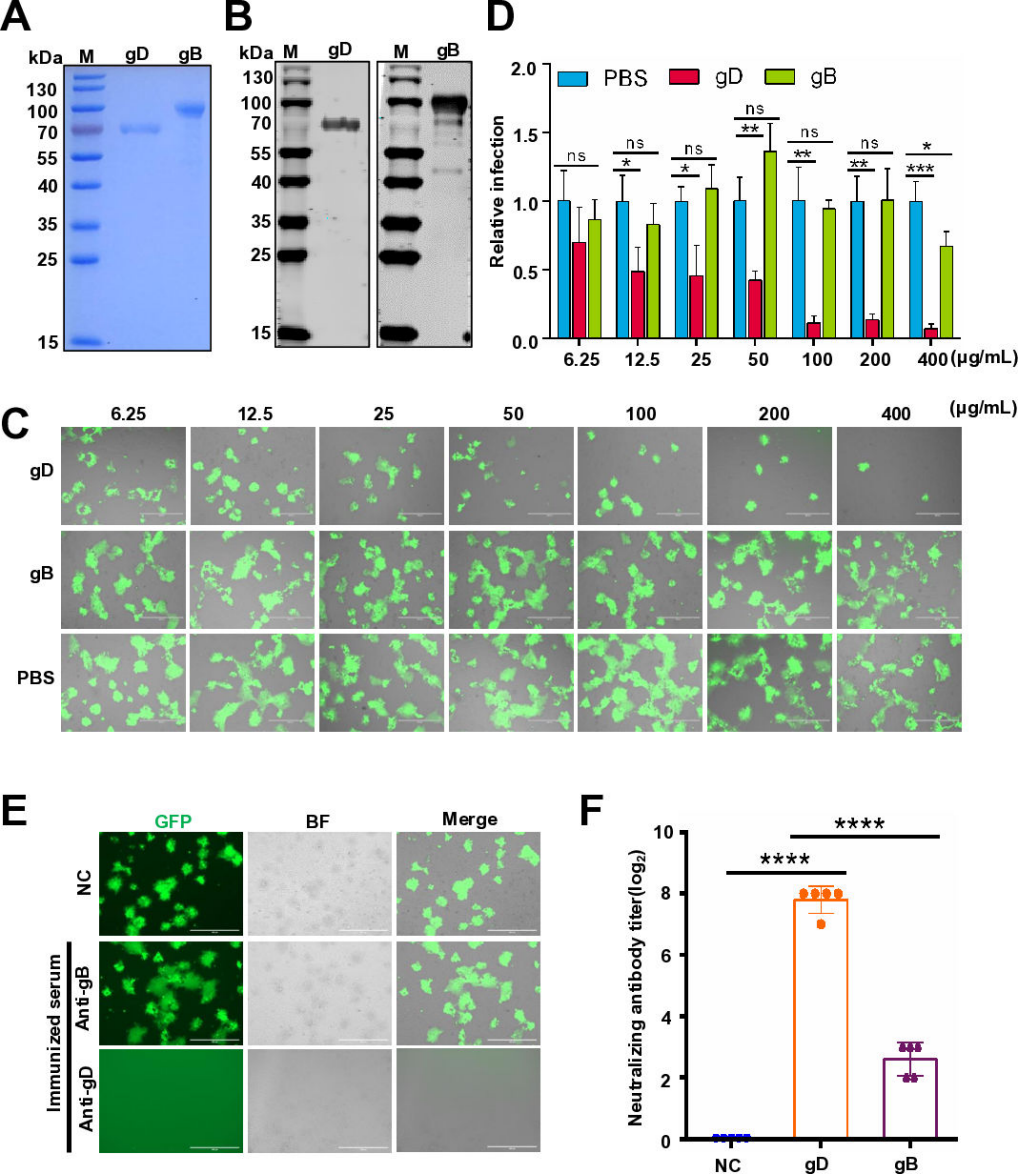
gD rather than gB induces robust neutralizing antibody. (**A**) Purified gB and gD proteins were subjected to SDS-PAGE. This experiment was conducted three times, and a representative result is presented. (**B**) Western blot for gB and gD with a specific antibody. This experiment was conducted three times, and a representative result is presented. (**C**) Viral inhibition effect mediated by indicated doses of gD or gB protein. Various concentrations of gB and gD were co-incubated with the PRV-EGFP reporter virus (multiplicity of infection = 0.01) on Vero E6 for 2 hours. Then washed three times and further cultured. PBS was used as the control. After 36 hours of infection, the infected cells were examined under a microscope. Scale bars, 400 µm. This experiment was conducted three times, and a representative result is presented. (**D**) The relative infection was determined by quantifying the area occupied by EGFP-positive cells using Image J software. The data represent the means ± standard deviation (SD) from three independent experiments. (**E**) Viral inhibition effect mediated by gB or gD immunized sera. Five mice per group were immunized, with vaccinations at weeks 0 and 2. Serum samples were collected 2 weeks after the booster. PRV-EGFP reporter virus (200 TCID_50_) was incubated with 50 µL of twofold serially diluted sera for 2 hours at 37°C. The mixtures were then transferred to a 96-well plate containing a monolayer of Vero E6 cells and incubated for an additional 2 hours at 37°C. After washing, the cells were cultured in DMEM supplemented with 2% FBS. Forty-eight hours post-infection, infected cells were examined under a microscope. Neutralization was observed when serum dilution reached a concentration of 1:16. Scale bars represent 400 µm. This experiment was conducted three times, and a representative result is presented. (**F**) Neutralizing antibodies were calculated using the Reed-Muench method at 48 h post-infection. The data represent the means ± standard deviation (SD) from five animals.

To identify the optimal antigenic target for PRV vaccine development, we first evaluated the immunogenicity of glycoproteins B (gB) and D (gD), which have been reported to contribute significantly to the induction of neutralizing antibodies (21, 22). We first expressed the gB and gD proteins in CHO cells. The expression of these proteins was confirmed via SDS-PAGE analysis and by using specific antibodies against gB and gD, respectively (Fig. 1A and B). We believe that an effective vaccine should stimulate the production of antibodies capable of inhibiting the virus’s interaction with its receptors, thereby neutralizing it. Our experiment aims to investigate the interactions between viral proteins gB and gD with cell receptors. Subsequently, we test which protein binds to and competes for these receptors. To this end, various concentrations of gB and gD were co-incubated with the PRV-EGFP reporter virus (23). It was observed that a high concentration of the gD protein effectively competes for receptor-binding sites and inhibits PRV replication in Vero E6 cells, whereas the impact of gB is minimal (Fig. 1C). The relative infection rate was quantified by measuring the EGFP-positive area, which demonstrated that the purified gD protein significantly suppressed PRV replication (Fig. 1D). Following this, individual mice were immunized with either gB or gD proteins. After the booster immunization, antibodies from the gD-immunized group demonstrated a significantly higher titer of neutralizing antibodies and completely inhibited PRV infection in Vero E6 cells. In contrast, the antibodies derived from the gB-immunized group exhibited a relatively lower titer of neutralizing antibodies (Fig. 1E and F). Based on these findings, we selected the viral gD gene as our mRNA target for subsequent experiments.

**Fig 2:**
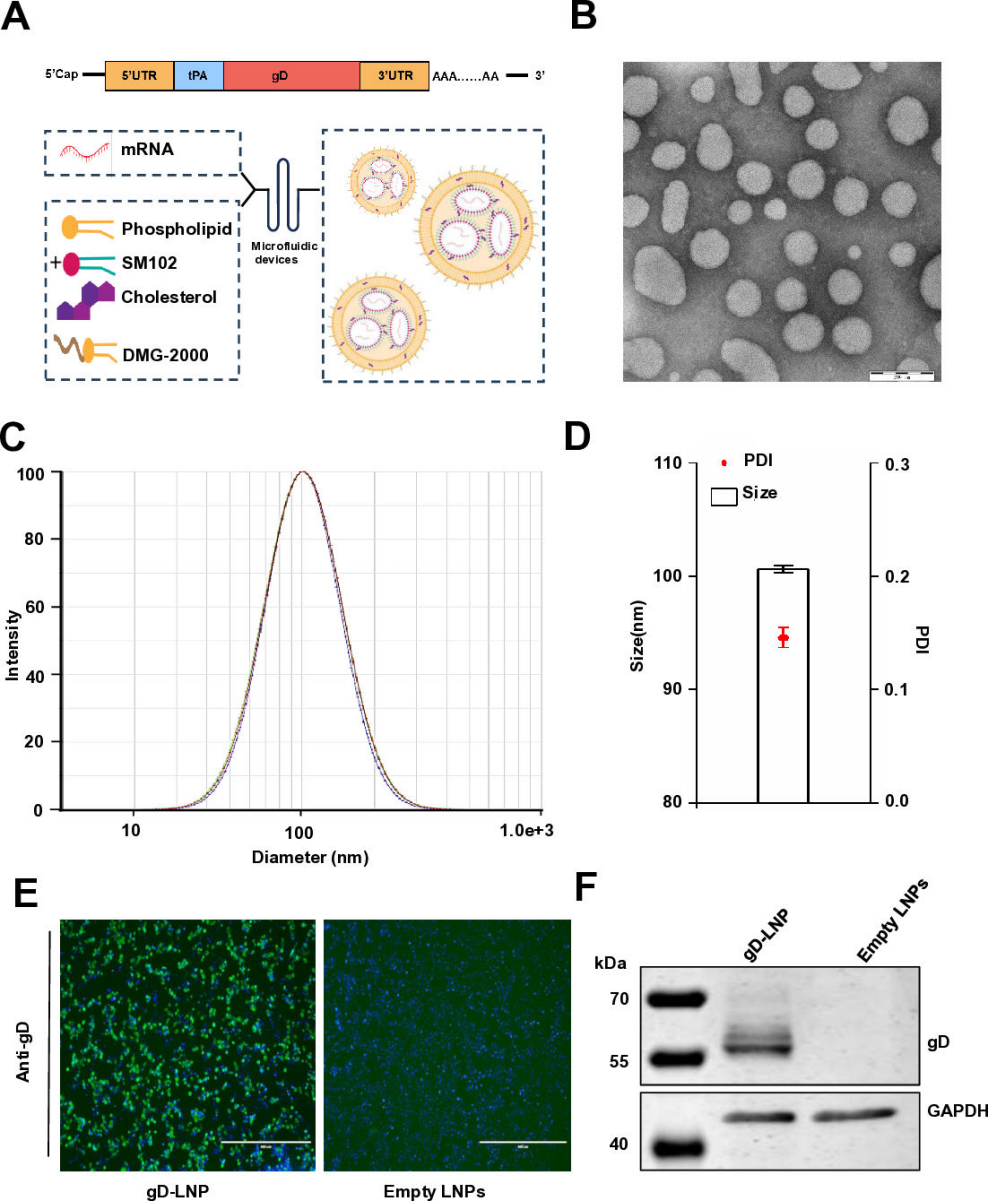
Design of gD mRNA and protein expression. (**A**) A flow chart demonstrates the process employed by a microfluidic device to prepare LNPs for the gD mRNA vaccine. Initially, cationic lipid (SM102), phospholipid with two stearoyl chains and a phosphorylcholine headgroup (DSPC), cholesterol, and DMG-2000 were dissolved in anhydrous ethanol at a mass ratio of 50:10:38.5:1.5, forming the organic phase. Concurrently, the gD mRNA containing the ectodomain (1–355 aa) of the PRV HeN1 strain, 5′-cap, as well as 5′ and 3′ UTRs was dissolved in citrate buffer solution (pH 4.0) to form the aqueous phase. Subsequently, the gD mRNA vaccine was produced by mixing these organic and aqueous phases. (**B**) A transmission electron microscopy (TEM) image provides a detailed visualization of the gD mRNA-LNPs. This experiment was conducted three times, and a representative result is presented. (**C**) gD mRNA transcripts were encapsulated within LNPs. The particle size of mRNA-LNPs and (**D**) PDI was evaluated. The data represent the means ± standard deviation (SD) from three independent experiments. (**E**) HEK293T cells were treated with 2 µg gD mRNA-LNPs. The empty LNPs served as the control group. After 24 hours of incubation, the expression of mRNA was analyzed by IFA and (**F**) Western blot with gD antibodies. GAPDH was used as a loading control. Scale bars, 400 µm. This experiment was conducted three times, and a representative result is presented.

The gD mRNA vaccine was designed according to the schematic shown in Fig. 2A. The gD ectodomain of PRV HeN1 variant strain was cloned into an mRNA vector containing a 5′-cap, as well as 5′ and 3′ UTRs. Furthermore, the tissue plasminogen activator signal sequence was incorporated to enhance the efficient secretion of gD proteins from eukaryotic cells. Subsequently, the gD mRNA transcripts were encapsulated within lipid nanoparticles (LNPs) comprising cationic lipid (SM102), phospholipid with two stearoyl chains and a phosphorylcholine headgroup (DSPC), cholesterol, and DMG-2000. The morphology of the mRNA vaccines is depicted in Fig. 2B. The resulting gD mRNA-LNPs formulations were evaluated for particle size, a critical parameter influencing delivery efficiency and biodistribution. Smaller particles can more readily penetrate tissues and cells, while larger particles may exhibit distinct pharmacokinetic properties. The optimal particle size range for effective delivery is generally between 50 and 200 nanometers. Furthermore, the polydispersity index (PDI), which indicates the uniformity of the particle size distribution, was assessed. A lower PDI value signifies a narrower size distribution, which is essential for ensuring consistent performance and safety. The results demonstrated that the gD mRNA-LNPs exhibited an average particle size of 100.62 nm with a narrow distribution characterized by a PDI of 0.146 (Fig. 2C and D). To further validate gD expression *in vitro*, we conducted an indirect immunofluorescence assay (IFA) (Fig. 2E) and Western blot analysis (Fig. 2F) after incubating HEK293T cells with gD mRNA-LNPs or Empty LNPs. The results demonstrated efficient expression of the gD protein, with GAPDH serving as a loading control.

### gD mRNA-LNPs induces sustained humoral and cellular immunity in mice

**Fig 3:**
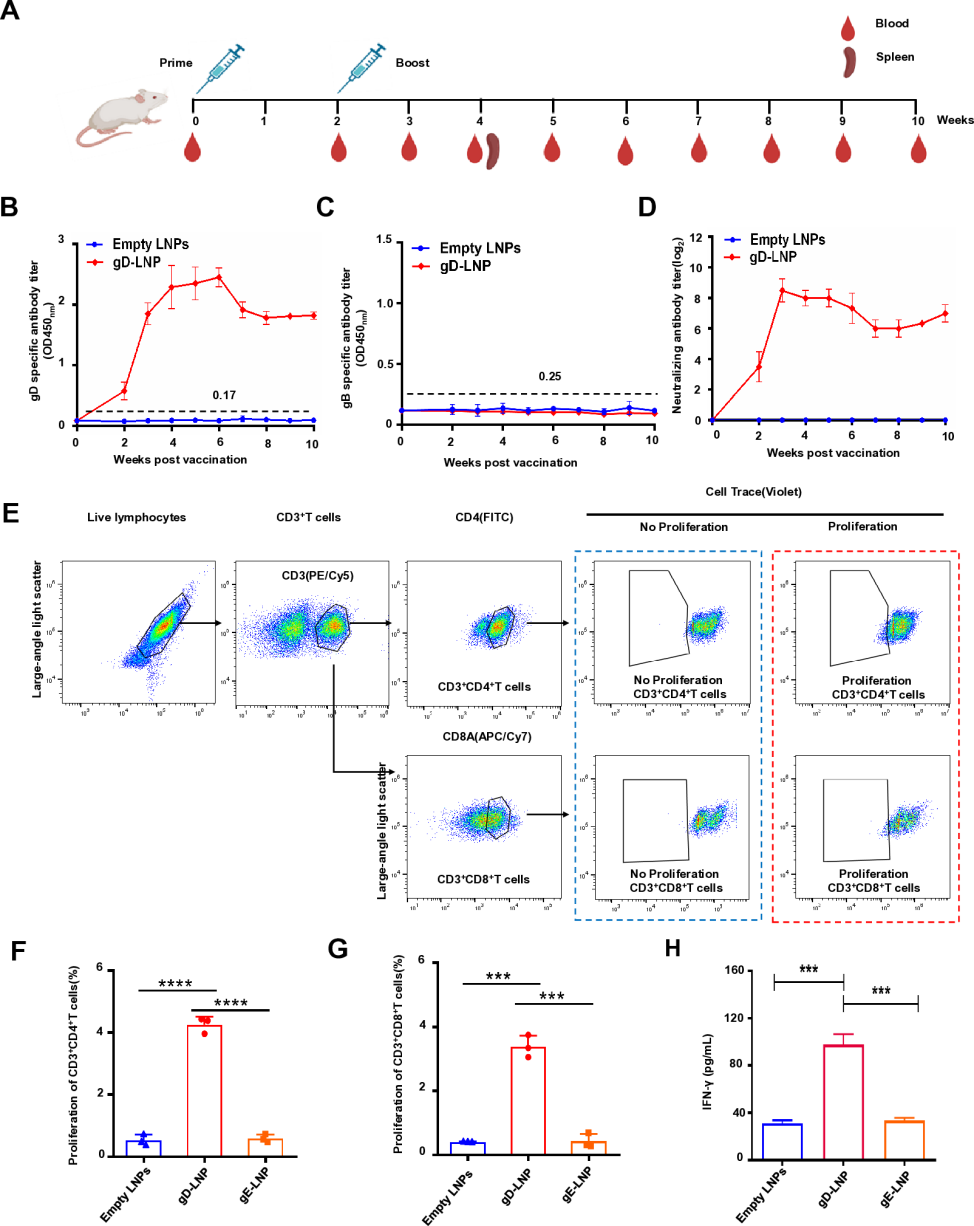
gD mRNA vaccine elicits high levels of PRV-specific humoral and T-cell response in mice. (**A**) Scheme of the animal experimental protocol. Six mice per group were immunized, with vaccinations at weeks 0 and 2. Serum samples were collected at indicated weeks after the booster. (**B**) PRV gD or (**C**) gB-specific antibodies in mice serum detected by indirect ELISA. The data represent the means ± standard deviation (SD) from three animals. (**D**) The PRV neutralizing antibody in mice serum induced by mRNA-LNPs was evaluated. PRV HeN1 (200 TCID_50_) was incubated with 50 µL of twofold diluted immunized serum on Vero E6 cells for 2 hours at 37°C. Subsequently, the mixtures were transferred to a 96-well plate containing monolayer Vero E6 cells and incubated for an additional 2 hours at 37°C. After washing the cells, neutralizing antibodies were quantified using the Reed-Muench method after a 48-hour infection period. The data represent the means ± standard deviation (SD) from three animals. (**E**) T-cell response elicited by gD mRNA and flow cytometry analysis of antigen-specific lymphocyte proliferation. Splenic lymphocytes isolated from mice at 28 days post-vaccination (dpv) were stimulated with gD mRNA-LNPs (10 µg), gE mRNA-LNPs (10 µg), or Empty LNPs at 37°C for 72 hours. The cells were subsequently stained with an anti-mouse CD3, CD4, and CD8 antibody, and flow cytometric analysis was conducted. Proliferation of CD3^+^CD4^+^ T cells (**F**), CD3^+^CD8^+^ T cells (**G**) for the indicated groups was calculated. The data represent the means ± standard deviation (SD) from three independent experiments. (**H**) IFN-γ production in mice vaccinated with gD mRNA vaccine. Mice spleen lymph single-cell suspensions were incubated with gD mRNA-LNPs (10 µg), gE mRNA-LNPs (10 µg), or Empty LNPs for 72 hours at 37°C. Subsequently, the cells were centrifuged, and the supernatants were collected to quantify IFN-γ levels using commercial mouse IFN-γ ELISA kits. The data represent the means ± standard deviation (SD) from three independent experiments.

We next evaluated the immunogenicity of gD mRNA vaccines in mice. A total of 12 mice were randomly divided into two groups (*n* = 6), with one group receiving immunization using a 10 µg dose of gD mRNA vaccine and subsequent boosting with an equivalent quantity of mRNA after 2 weeks from the initial immunization. Another group was designated as the control group and received empty LNPs for vaccination (Negative control, NC). All mice were vaccinated via intramuscular injection. Serum samples were collected at different time points for PRV antibody detection (Fig. 3A). The presence of PRV gD-specific antibodies was detected using indirect ELISA, and the results demonstrated that the gD mRNA immunized group exhibited significantly elevated levels of PRV gD-specific antibodies, which persisted for at least 10 weeks post-vaccination (Fig. 3B). In contrast, no detectable PRV gD-specific antibodies were observed in the control group (Fig. 3B). Additionally, no detectable PRV gB-specific antibodies were observed in the gD mRNA immunized group or control group using indirect ELISA (Fig. 3C). Collectively, these data suggest that the gD mRNA vaccine elicited a robust-specific immune response against gD. Furthermore, a high level of PRV neutralizing antibody was elicited by this vaccine as evidenced by the detected neutralizing antibody titer in the gD mRNA immunized group (Fig. 3D).

To evaluate the T-cell response stimulated by gD mRNA, mouse splenic lymphocytes were isolated and subjected to flow cytometry analysis of antigen-specific lymphocyte proliferation. Therefore, CD3^+^ CD4^+^ T-cells and CD3^+^ CD8^+^ T-cells were gated for analysis of antigen-specific lymphocyte proliferative responses (Fig. 3E). Subsequently, these lymphocytes were stimulated *in vitro* with gD mRNA-LNPs (10 µg) or empty LNPs. Additionally, we employed gE mRNA-LNPs (10 µg), another viral gene of PRV, as a more rigorous control. The results demonstrated that the groups immunized with gD mRNA exhibited a significantly higher rate of CD3^+^ CD4^+^ T-cell and CD3^+^ CD8^+^ T-cell proliferation compared to the control group (Fig. 3F and G). We further confirmed cellular immune responses by measuring IFN-γ secretion from lymphocytes stimulated with gD mRNA-LNPs. It was observed that lymphocytes from mice vaccinated with gD mRNA exhibited significantly higher levels of IFN-γ production compared to controls, which were stimulated with empty LNPs (Fig. 3H). These findings suggest that gD mRNA elicits robust PRV-specific humoral and T-cell responses in mice.

### Complete protection against high-dose PRV challenge in mice

**Fig 4:**
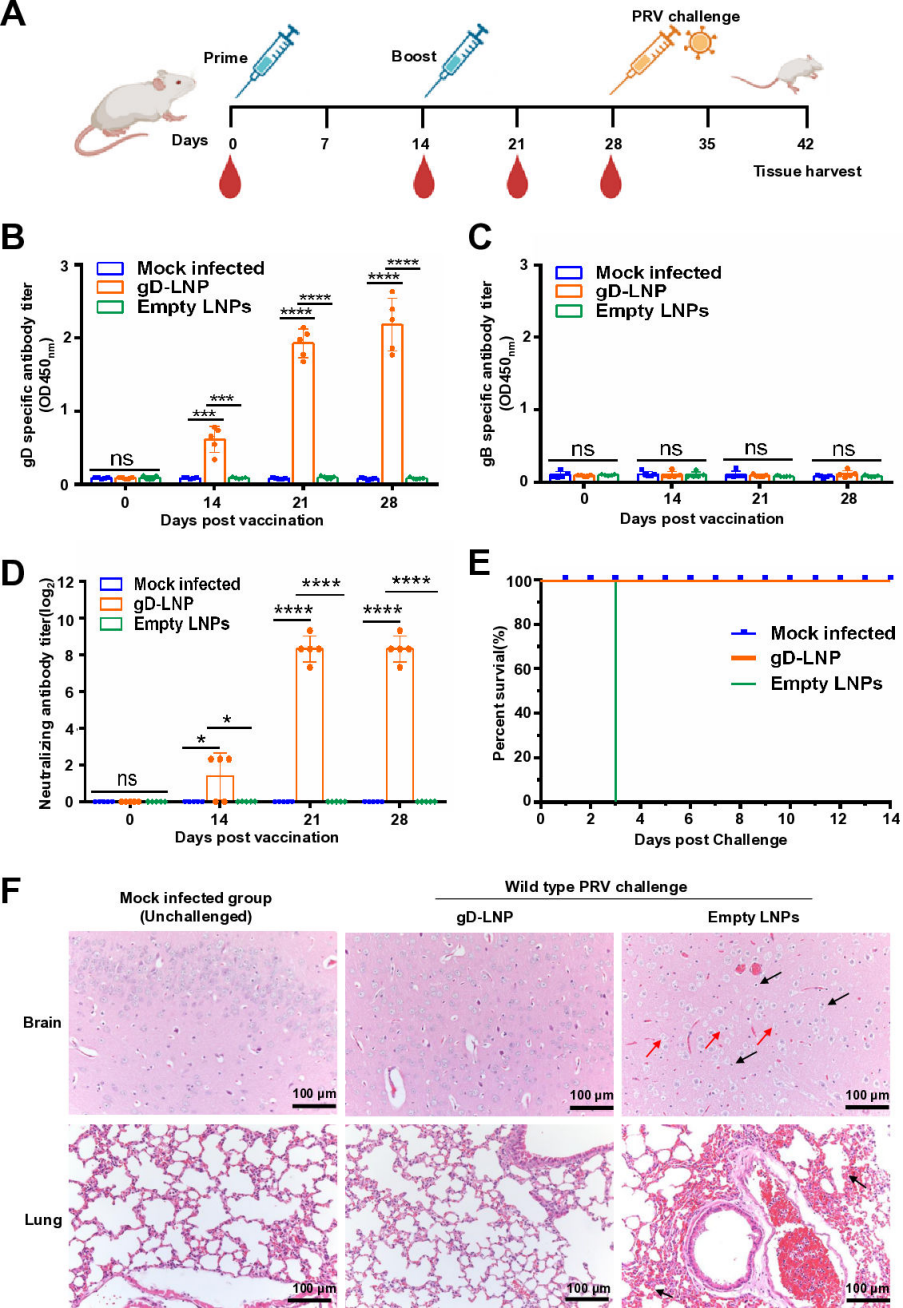
gD mRNA vaccine protects mice against PRV challenge. (**A**) Scheme of the animal experimental protocol. Five mice per group were immunized, with vaccinations at weeks 0 and 2. Serum samples were collected at indicated weeks after the booster. PRV gD-specific antibodies (**B**) and gB-specific antibodies (**C**) in mice serum immunized by gD mRNA vaccine or empty LNPs by indirect ELISA. The data represent the means ± standard deviation (SD) from five animals. (**D**) PRV neutralizing antibody in mice serum induced by mRNA-LNPs. PRV HeN1 (200 TCID_50_) incubated with 50 µL of twofold diluted immunized serum for 2 hours at 37°C. Subsequently, the mixtures were transferred to a 96-well plate containing monolayer Vero E6 cells and incubated for an additional 2 hours at 37°C. After washing the cells, neutralizing antibodies were quantified using the Reed-Muench method after a 48-hour infection period. The data represent the means ± standard deviation (SD) from five animals. (**E**) The survival rate of mice immunized with mRNA-LNPs or empty LNPs after challenge. (**F**) Hematoxylin and eosin (HE) staining of brain and lung tissues. Microscopic lesions in the brain and lung from different groups following PRV challenge are shown. Histopathological changes induced by the PRV HeN1 strain are indicated by arrows. In the brain, necrosis of neuronal cells in the cerebral cortex (indicated by the red arrow) and mild infiltration of glial cells (indicated by the black arrow) were observed. In the lungs, mild congestion was noted in the capillaries of the pulmonary alveolar walls (indicated by the black arrow). Scale bars, 100 µm. This experiment was conducted three times, and a representative result is presented.

We next investigated the protective efficacy of gD mRNA vaccine against PRV challenge in mice (Fig. 4A). The gD mRNA group received two doses of the gD mRNA vaccine, each at a dosage of 10 µg via intramuscular injection. Our results demonstrated that the gD mRNA immunized group exhibited significantly elevated levels of PRV gD-specific antibodies (Fig. 4B and C) and neutralizing antibodies for the PRV HeN1 strain (Fig. 4D). Next, mice were challenged with the wild-type PRV HeN1 strain via intramuscular injection. In this study, we challenged mice with a high dose of wild-type PRV (2 × 10^4^ PFU). We observed that all mice immunized with the gD mRNA vaccine survived after challenge; however, the empty LNPs immunized group experienced 100% mortality following challenge (Fig. 4E). The clinical signs were assessed using clinical scores (Fig. S1). Additionally, viral loads in indicated tissues were detected, revealing significantly lower viral copies in the gD mRNA vaccine group compared to the empty LNPs-immunized group (Fig. S2). Furthermore, histopathological changes in the brain and lungs were evaluated. Our findings indicated that no histopathological alterations were observed in either the gD mRNA vaccine group or the negative control group. However, the group immunized with empty LNPs exhibited significant microscopic lesions in both the brain and lungs. Specifically, the alterations observed in the brains of mice treated with empty LNPs are characterized by glial cell proliferation and marked degeneration of neuronal cells (Fig. 4F). In the lungs of mice administered empty LNPs, congestion and infiltration by inflammatory cells lead to widening of alveolar septa, accompanied by partial degeneration and necrosis of bronchial epithelial cells. Additionally, fibrinous exudate is present within the alveolar spaces (Fig. 4F). Overall, the gD mRNA vaccine provided full protection against PRV challenge.

### gD mRNA vaccine provides protection against PRV infection in mice via respiratory route

**Fig 5:**
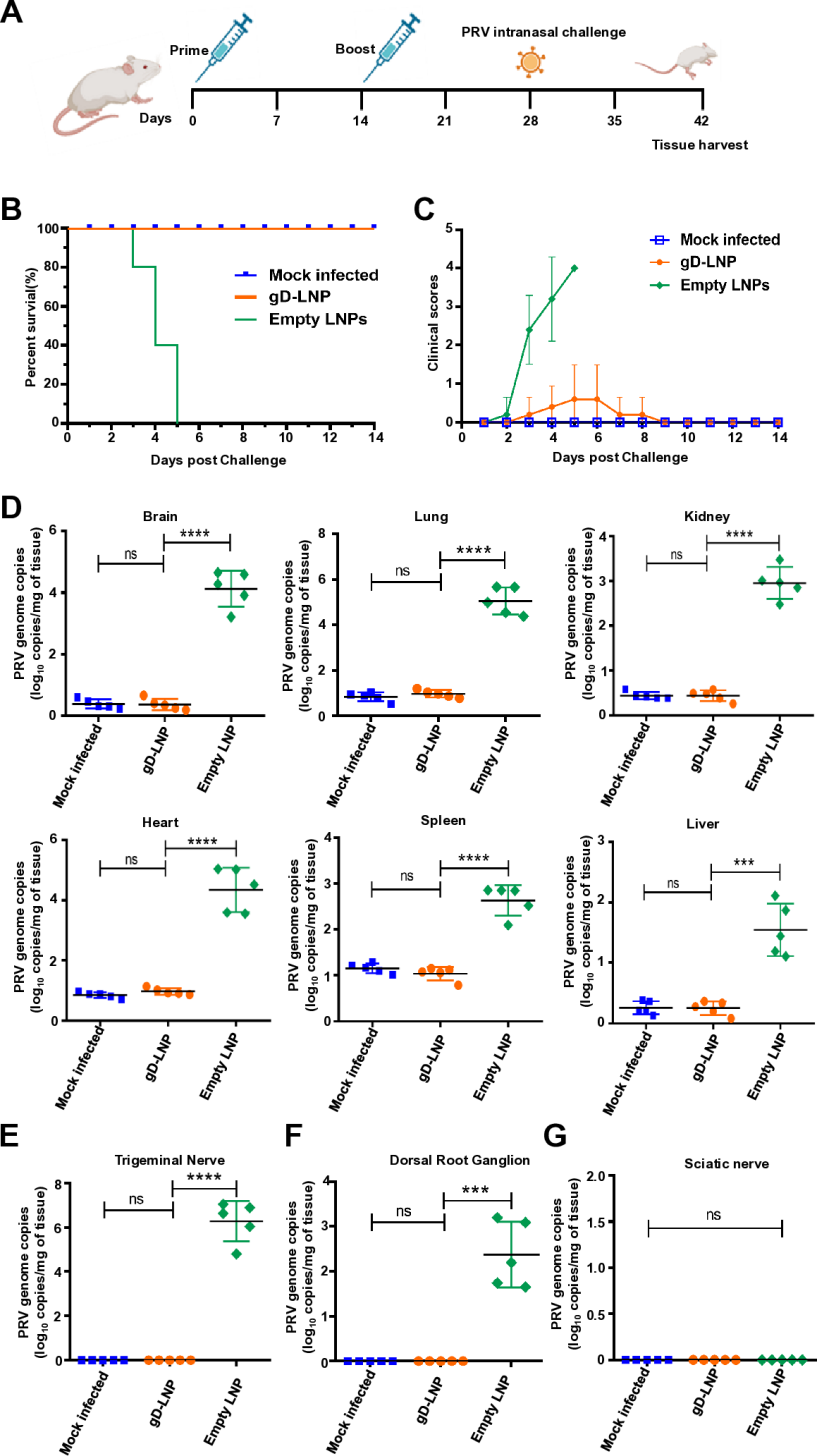
gD mRNA protects mice against PRV challenge via the respiratory route challenge. (**A**) Experimental protocol overview: Five mice per group were immunized with vaccinations at weeks 0 and 2. Mice were challenged via the intranasal route 2 weeks after the booster. (**B**) The survival rate of mice immunized with gD mRNA-LNPs or empty LNPs after intranasal challenge. (**C**) The clinical score of mice immunized with gD mRNA-LNPs or empty LNPs after intranasal challenge. (**D**) The viral loads quantified by real-time PCR in the indicated mouse tissues, including brain, lung, kidney, heart, spleen, and liver. The data represent the means ± standard deviation (SD) from five animals. (**E**) The viral loads quantified by real-time PCR in the trigeminal nerve of mice immunized with gD mRNA-LNPs or empty LNPs after intranasal challenge. The data represent the means ± standard deviation (SD) from five animals. (**F**) The viral loads quantified by real-time PCR in the dorsal root ganglion of mice immunized with gD mRNA-LNPs or empty LNPs after intranasal challenge. The data represent the means ± standard deviation (SD) from five animals. (**G**) The viral loads quantified by real-time PCR in the sciatic nerve of mice immunized with gD mRNA-LNPs or empty LNPs after intranasal challenge. The data represent the means ± standard deviation (SD) from five animals.

PRV typically spreads through the respiratory route. Therefore, we subsequently evaluated whether this vaccine induces mucosal protection. Following two rounds of immunization, we challenged the vaccinated mice with wild-type PRV (1 × 10^3^ PFU) via the intranasal route (Fig. 5A). The results demonstrated that all mice immunized with the gD mRNA vaccine survived following the intranasal challenge. In contrast, the group that received empty LNPs exhibited a 100% mortality rate after undergoing the same intranasal challenge (Fig. 5B). The clinical signs were further evaluated using clinical scores (Fig. 5C). Additionally, we assessed viral loads in the specified tissues. The results indicated a significantly lower number of viral copies in the gD mRNA vaccine group compared to the empty LNPs immunized group (Fig. 5D). Due to the potential for PRV infection to induce latent infections within the nervous system, we also evaluated viral loads in the trigeminal nerve, dorsal root ganglion, and sciatic nerve of mice that were immunized with gD mRNA-LNPs or empty LNPs following intranasal challenge. The results indicated that the gD mRNA vaccine provided complete protection against PRV intranasal challenge (Fig. 5E through G).

### gD mRNA vaccine elicits high levels of PRV-specific humoral and T-cell response in piglets post vaccination

**Fig 6:**
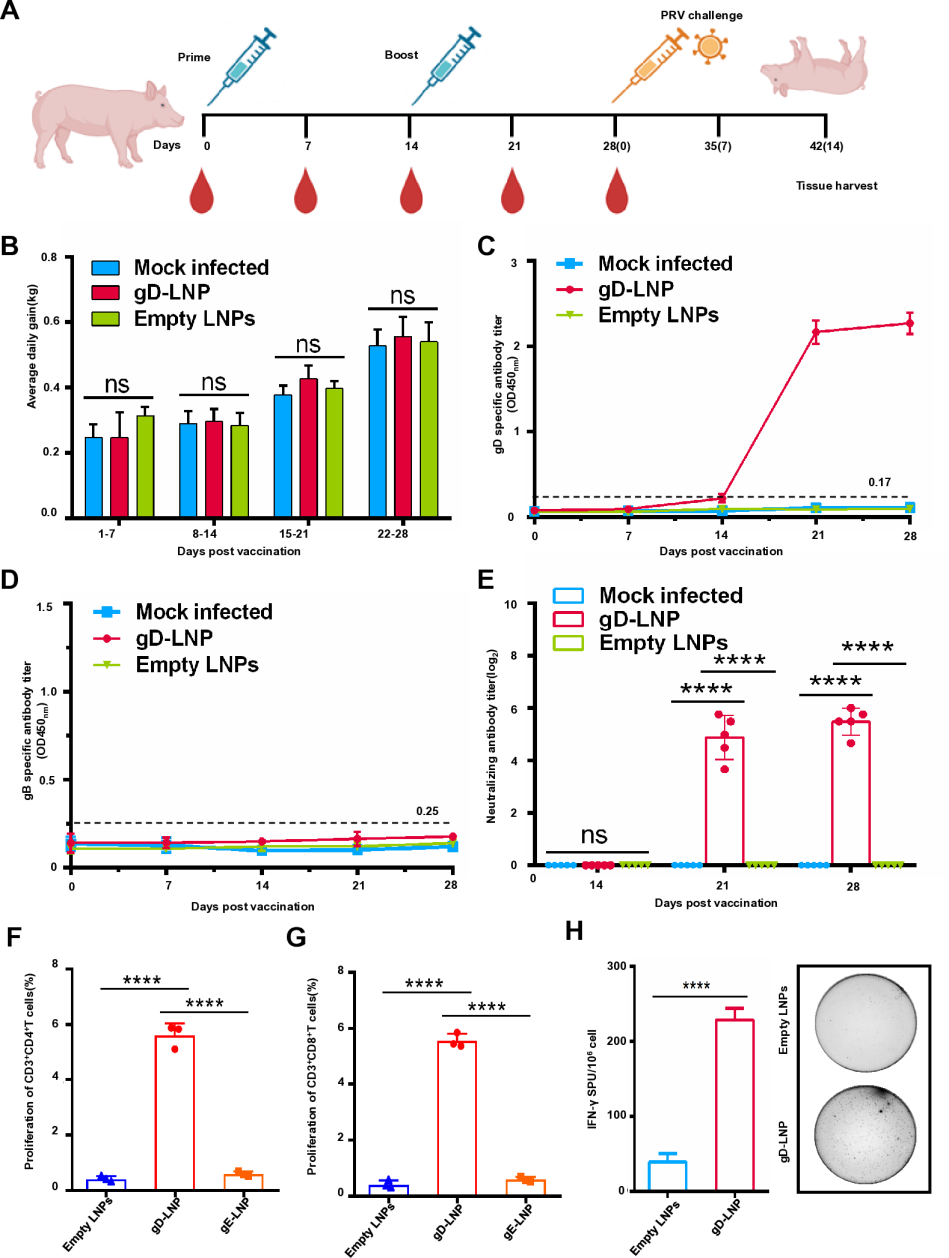
gD mRNA vaccine elicits high levels of PRV-specific humoral and T-cell response in piglets. (**A**) Scheme of the animal experimental protocol. Five piglets per group were immunized, with vaccinations at weeks 0 and 2. Serum samples were collected at indicated weeks after the booster. (**B**) The average daily weight gain between the group immunized with gD mRNA vaccines and controls. The data represent the means ± standard deviation (SD) from five animals. (**C**) PRV gD-specific antibodies in piglets’ serum detected by indirect ELISA. The data represent the means ± standard deviation (SD) from five animals. (**D**) PRV gB-specific antibodies in piglets’ serum detected by indirect ELISA. The data represent the means ± standard deviation (SD) from five animals. (**E**) PRV-neutralizing antibody in piglets’ serum induced by mRNA-LNPs. PRV HeN1 (200 TCID_50_) incubated with 50 µL of twofold diluted immunized swine serum on for 2 hours at 37°C. Subsequently, the mixtures were transferred to a 96-well plate containing monolayer Vero E6 cells and incubated for an additional 2 hours at 37°C. After washing the cells, neutralizing antibodies were quantified using the Reed-Muench method after a 48-hour infection period. The data represent the means ± standard deviation (SD) from five animals. (**F**) and (**G**) T-cell response stimulated by gD mRNA-LNPs and flow cytometry analysis of antigen-specific lymphocyte proliferation. Peripheral blood lymphocytes from piglets were isolated and stained with CellTrace Violet Cell Proliferation Kit. Subsequently, 1,000,000 stained cells were stimulated with gD mRNA-LNPs (10 µg) at 37°C with 5% CO2 for 72 hours. Empty LNPs stimulated cells were used as negative controls. The cells were then stained with anti-porcine CD3, CD4, and CD8 antibodies. After three washes with PBS, flow cytometric analysis was performed. The data represent the means ± standard deviation (SD) from three independent experiments. (**H**) IFN-γ production in piglets vaccinated with gD mRNA vaccine. The cellular immune responses in the vaccinated piglets were evaluated using IFN-γ enzyme-linked immunospot (ELISPOT). Briefly, 300,000 PBMCs were stimulated with gD mRNA-LNPs (5 µg), or empty LNPs, followed by incubation at 37°C for 48 hours. Empty LNPs stimulated cells were used as negative controls. The plates were washed and then incubated with a detection antibody. Streptavidin-HRP was added to the plates and incubated. After final washes, the TMB substrate solution was applied and subsequently stopped with water. The air-dried plates were analyzed using an ELISPOT reader to determine the number of spot-forming cells per million cells. The data represent the means ± standard deviation (SD) from three independent experiments.

We next evaluated the immunogenicity of gD mRNA vaccines in piglets. The experimental timeline and design are schematically illustrated in Fig. 6A. There was no significant difference observed in the average daily weight gain between the group immunized with gD mRNA vaccines and the control group which was vaccinated with empty LNPs (Fig. 6B). Additionally, gD-specific antibodies were detectable 14 days post-vaccination (Fig. 6C). As a control, we also assessed the presence of gB antibodies; however, no gB antibodies were detected (Fig. 6D). Moreover, a robust PRV neutralizing antibody response was elicited at day 21 after gD mRNA immunization (Fig. 6E). To characterize cellular immunity, we performed lymphocyte proliferation assays (Fig. S3). Flow cytometric analysis showed significantly enhanced CD3^+^CD4^+^ T-cell and CD3^+^CD8^+^ T-cell proliferation indices in vaccinated piglets compared to controls (Fig. 6F and G). Furthermore, we confirmed cellular immune responses by measuring IFN-γ secretion from lymphocytes stimulated with gD mRNA-LNPs. Notably, piglets vaccinated with gD mRNA exhibited significantly elevated levels of IFN-γ production compared to control piglets (Fig. 6H). These findings indicate that in piglets, gD mRNA vaccine elicits potent PRV-specific humoral and T-cell responses.

### gD mRNA vaccine prevents clinical disease and viral shedding in piglets

**Fig 7:**
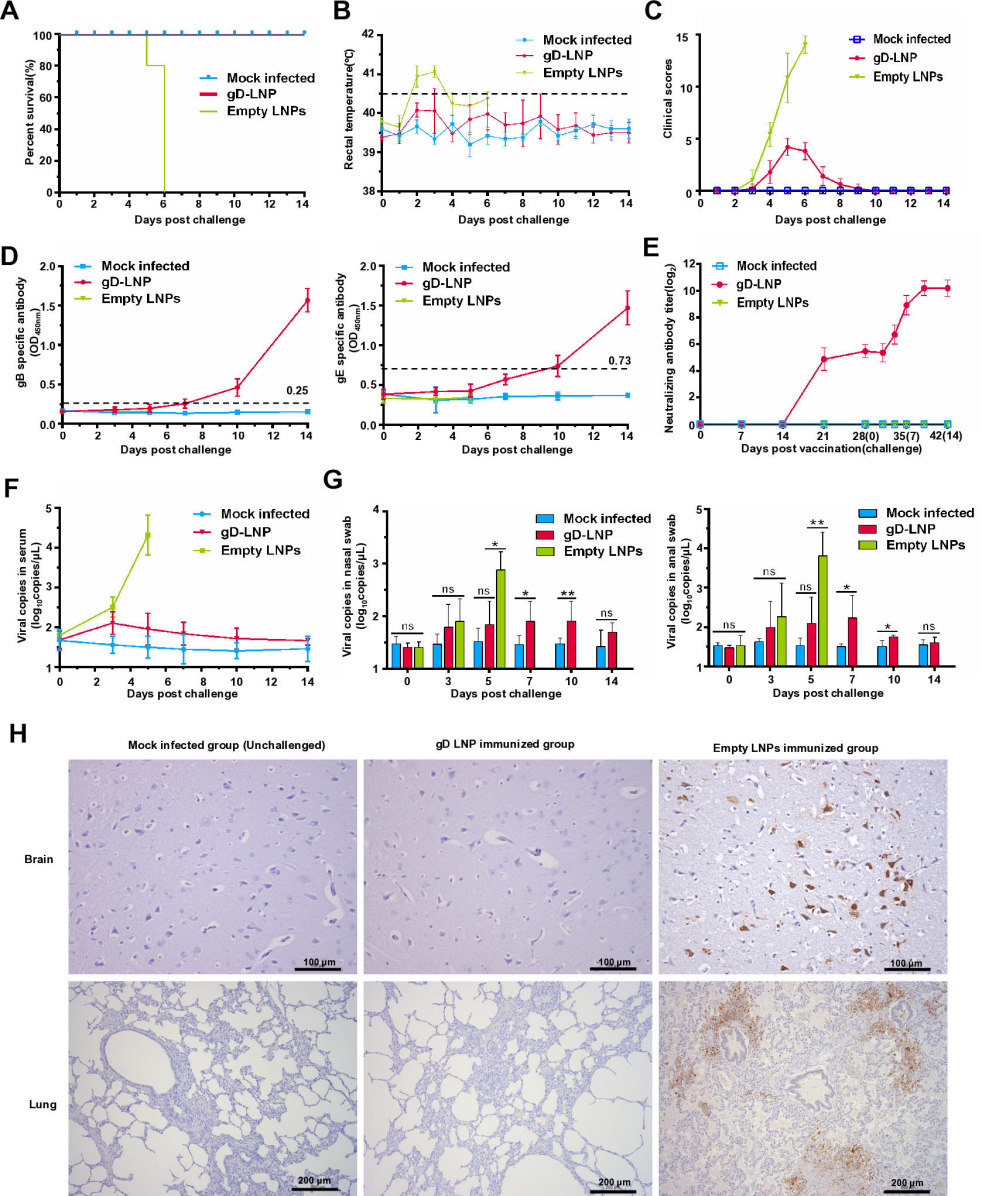
gD mRNA vaccine protects piglets against PRV challenge. (**A**) The survival rate of piglets immunized with mRNA-LNPs after wild-type PRV challenge. (**B**) The rectal temperature and (**C**) clinical signs of the gD mRNA vaccine-immunized group and the control group. (**D**) gB-specific antibodies and gE-specific antibodies with gD mRNA vaccine-immunized group and control group after wild-type PRV challenge. The data represent the means ± standard deviation (SD) from five animals. (**E**) PRV-neutralizing antibody level in piglets’ serum immunized with mRNA-LNPs and PRV neutralizing antibody level after wild-type PRV challenge. The data represent the means ± standard deviation (SD) from five animals. (**F**) The viral loads in serum in the gD mRNA vaccine group and the control group. The data represent the means ± standard deviation (SD) from five animals. (**G**) The viral copies in nasal and anal swabs in the gD mRNA vaccine group and the control group. The data represent the means ± standard deviation (SD) from five animals. (**H**) Immunohistochemistry analysis of brain and lung tissues of different groups following PRV challenge. Scale bars of the brain, 100 µm. Scale bars of lung, 200 µm. This experiment was conducted three times, and a representative result is presented.

We next investigated the protective efficacy of gD mRNA vaccine against PRV challenge in piglets. gD mRNA-LNPs-immunized group and the empty LNPs-vaccinated group were challenged with wild-type PRV HeN1 strain. The mock-infected group, serving as a negative control, was not exposed to the virus during the experimental period. It was observed that all piglets immunized with the gD mRNA vaccine survived after challenge; however, the empty LNPs-vaccinated group experienced 100% mortality following challenge (Fig. 7A). The rectal temperature and clinical signs were recorded (Fig. 7B and C).

Furthermore, gB-specific antibodies and gE-specific antibodies were detectable 10 days post-challenge in the group vaccinated with gD mRNA vaccine (Fig. 7D). Interestingly, there was a significant increase in neutralizing antibodies in the group vaccinated with gD mRNA-LNPs post-challenge (Fig. 7E). Furthermore, viral loads in serum were assessed and demonstrated lower viral copies in the gD mRNA vaccine group compared to the empty LNPs-vaccinated group (Fig. 7F). Additionally, virus shedding was evaluated by detecting viral copies in nasal and anal swabs, revealing a lower level of virus shedding in the gD mRNA vaccine group (Fig. 7G). Moreover, viral loads in indicated tissues were also evaluated, demonstrating lower viral loads in the gD mRNA vaccine group compared to the empty LNPs-vaccinated group (Fig. S4). Furthermore, histopathological changes in the brain and lungs were examined, with no significant histopathological alterations observed in the gD mRNA vaccine group, which were similar to those in the mock-infected group (Fig. S5). However, severe microscopic lesions were observed in the brain and lungs of piglets challenged with wild-type PRV in the empty LNPs-vaccinated group (Fig. S5). In pigs treated with empty LNPs, notable changes in brain tissue include proliferation of glial cells, extensive degeneration and necrosis of neuronal cells, presence of vascular cuffs, as well as occasional necrosis among glial cells. The alterations identified in the lungs of pigs receiving empty LNPs encompass congestion, significant perivascular edema, infiltration by inflammatory cells, along with fibrinous exudate within the alveolar lumen that is associated with necrotic shedding from alveolar epithelial cells (Fig. S5). The results of immunohistochemistry indicate that, in the gD mRNA vaccine group, viral antigens were undetectable (Fig. 7H). Overall, the gD mRNA vaccine provided complete protection against PRV challenge in piglets.

### gD mRNA vaccine elicits cross-neutralizing antibodies against diverse PRV strains

**Fig 8:**
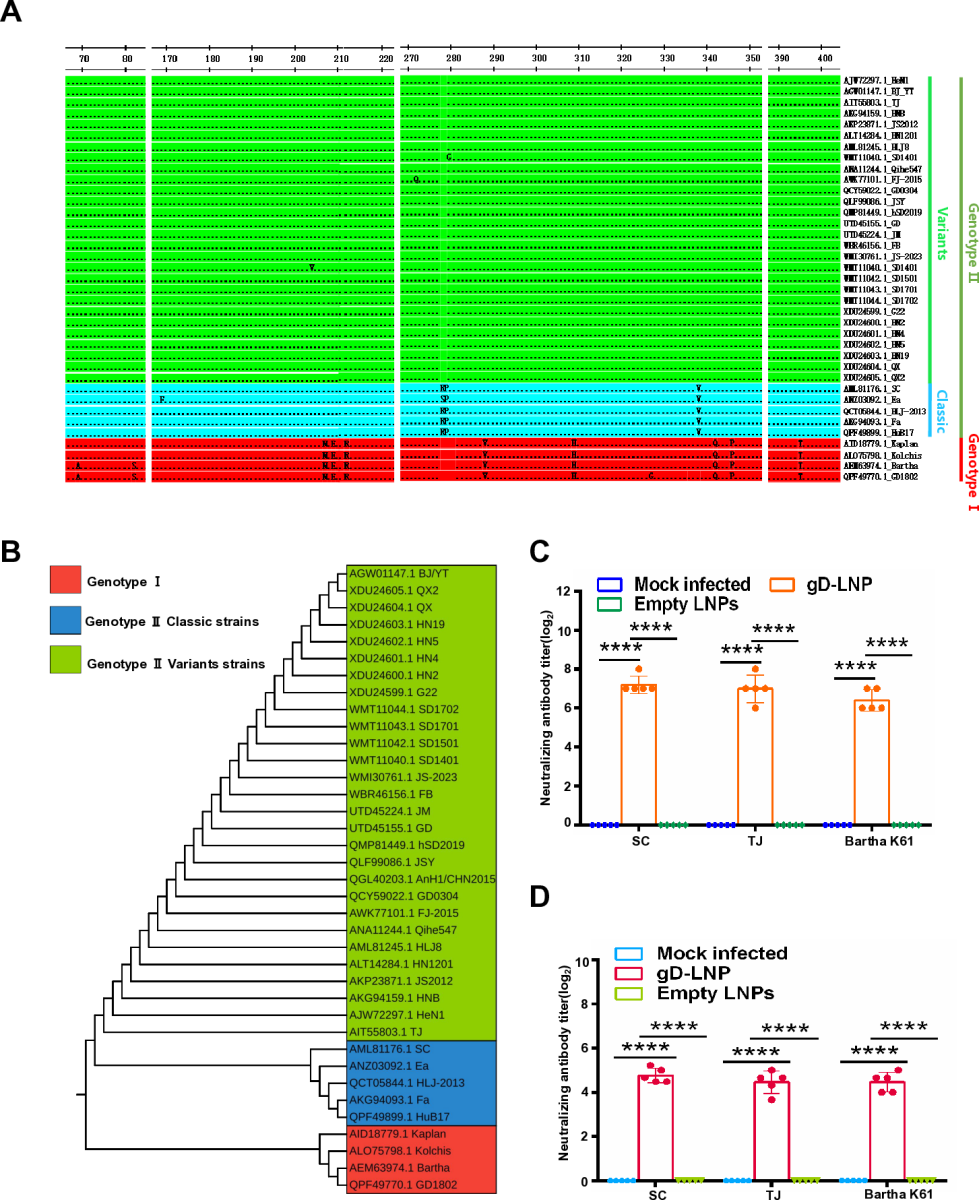
gD mRNA vaccine elicits cross-neutralizing antibodies against diverse PRV strains. (**A**) The amino acid sequences of the gD protein from the specified PRV strains were compared using MegaAlign software. (**B**) Phylogenetic analysis of the gD protein from these PRV strains was conducted with MEGA software. The neutralizing antibodies present in serum samples from vaccinated mice (**C**) and piglets (**D**) were assessed against representative PRV strains: SC, TJ, and Bartha K61. The data represent the means ± standard deviation (SD) from five animals.

To evaluate the breadth of humoral immunity induced by the gD mRNA vaccine, sera collected at 28 days post-vaccination (dpv) from immunized mice and piglets were analyzed for cross-reactivity and neutralizing activity against heterologous PRV strains. Initially, we evaluated the conservation of gD amino acid sequences across various PRV strains. We retrieved 37 gD amino acid sequences from the NCBI GenBank database, which included 4 sequences from genotype I, 5 sequences from genotype II classic strains, and 28 sequences from genotype II variant strains. Subsequently, we employed MegaAlign software to analyze the conservation of the gD protein’s amino acid sequence among different PRV strains. The results indicated that the sequence of the PRV gD protein is conserved, with only a few amino acids exhibiting mutations across all aligned sequences (Fig. 8A and B). To evaluate the cross-reactivity and broad-spectrum neutralizing activity elicited by the gD mRNA vaccine, we selected HeN1, SC, TJ, and Bartha K61 from various branches within the phylogenetic tree. IFA revealed that sera from both gD mRNA-vaccinated mice (Fig. S6A) and piglets (Fig. S6B) exhibited robust cross-reactivity with four genetically distinct PRV strains: HeN1, SC, TJ, and Bartha K61. Neutralization assays further demonstrated broad-spectrum protective potential. Serum from vaccinated mice (Fig. 8C) and piglets (Fig. 8D) effectively neutralized SC, TJ, and Bartha K61 strains, with neutralization titers comparable to those observed against the homologous HeN1 strain. This cross-neutralizing activity correlated with the conserved epitope recognition profile observed in IFA, confirming that gD mRNA immunization elicits antibodies targeting conserved antigenic regions across divergent PRV strains.

## DISCUSSION

In this study, we have demonstrated the safety and efficacy of an mRNA vaccine against PRV in both mice and piglets. Additionally, our study has shown that these vaccines not only induce potent neutralizing antibodies but also stimulate robust specific T-cell responses. Indeed, several DNA vaccines have been developed utilizing plasmid DNA encoding the gB, gC, gD, or gE proteins of PRV; however, these vaccines have demonstrated a moderate level of protection in pigs against PRV infection (24, 25). In our study, we demonstrated that the gD subunit protein elicited a robust humoral response (Fig. 1F). We think there are two primary advantages of the gD mRNA-LNPs vaccine compared to the gD subunit protein vaccine. First, the gD mRNA-LNPs can effectively enter host cells and express endogenous-like proteins, which can more efficiently stimulate T-cell responses. Second, in the event of a new emergence of PRV, developing a new gD subunit protein vaccine requires a significant amount of time; conversely, mRNA vaccines can be produced much more rapidly, enabling a swift response to emerging viral threats. In fact, for PRV infection, both neutralizing antibodies and T-cell responses are essential components of vaccine-mediated protection. Neutralizing antibodies primarily target extracellular viral particles, effectively preventing the spread of the virus; however, their impact on intracellular viruses is limited (26). Conversely, T-cell immune responses—particularly those mediated by CD8 T cells—are capable of eliminating infected target cells, thereby reducing potential sites for viral replication. It has been reported that both gB and gD contribute significantly to the induction of neutralizing antibodies (21, 22). The primary role of gD is to directly interact with cellular receptors and other cellular molecules, facilitating the attachment and entry of PRV (27, 28). Meanwhile, gB primarily facilitates fusion between the viral envelope and the host cell membrane, thereby nucleocapsids and tegument proteins are released into the host cell cytoplasm for subsequent replication (29). Our current study demonstrates that gD elicits a robust and high level of neutralizing antibodies, in contrast to gB. While purified soluble gD and gB, overexpressed in CHO cells, exhibit differential abilities to induce neutralizing antibodies, this observation does not preclude the potential of gB mRNA-based vaccines to provide protection. Furthermore, the gB antigen may be effective in inducing T-cell-mediated immunity or potentially facilitating the production of non-neutralizing antibodies. These antibodies have demonstrated significant efficacy in mediating effector functions such as antibody-dependent cellular cytotoxicity and antibody-dependent cellular phagocytosis. Additionally, our recent findings suggest that except for gB, other viral antigens may be critical for PRV protection (30). We utilized anticodon-engineered transfer RNAs to generate live but replication-defective viruses lacking gB expression, which still provided full protection against PRV challenge (30). In fact, the levels of neutralizing antibodies induced by this experiment were also higher than those stimulated by replication-defective vaccines (30). However, immunization with replication-defective viruses in mice still provides adequate immune protection, indicating that T-cell immune responses are also crucial for immune protection against PRV in mice (31). In our study, we also demonstrated that the gD mRNA vaccine effectively stimulated a robust T-cell response.

PRV has been successfully eradicated in several countries, primarily through the implementation of vaccine-based serological diagnostics (3). Consequently, the gD mRNA vaccine becomes more feasible using serological diagnostics to differentiate from PRV infection. This vaccine will accelerate the global eradication of PRV. Therefore, it is helpful to develop a serological diagnostic method targeting gD in the future. Furthermore, by examining antibodies against other viral proteins, it becomes possible to differentiate between immune responses resulting from gD mRNA vaccination and those arising from wild virus infection. In conclusion, we developed a gD mRNA-LNPs vaccine, and this vaccine provides protective immunity against PRV challenge in both mice and piglets and elicits high levels of neutralizing antibodies and specific T-cell responses, indicating its potential as a new and promising vaccine candidate for the fight against PRV.

## MATERIALS AND METHODS

### Cells, viruses, proteins, and antibodies

Human embryonic kidney cells (HEK293T, ATCC CRL-11268) and African green monkey kidney cells (Vero-E6, ATCC CRL-1586) were cultured in Dulbecco’s modified Eagle’s medium (DMEM, Gibco, USA) supplemented with 10% fetal bovine serum (FBS, Excell, Australia) at a temperature of 37°C with a CO_2_ concentration of 5%. The PRV HeN1 strain (GenBank Accession No. KP098534), SC strain (GenBank Accession No. KT809429), TJ strain (GenBank Accession No. KJ789182), and Bartha K61 strain (GenBank Accession No. JF797217) were stored at −80°C in our laboratory. The PRV HeN1 EGFP strain was maintained as described in our previous report (29, 32). The Anti-GAPDH antibody was procured from Proteintech (Proteintech, China). PE/Cyanine5 anti-mouse CD3 antibody and APC/Cyanine7 anti-mouse CD8a antibody were procured from Biolegend (Biolegend, USA). The FITC rat anti-mouse CD4 antibody was sourced from BD-Pharmingen (BD-Pharmingen, USA). Mouse anti-porcine CD3E-SPRD antibody, mouse anti-porcine CD4-FITC antibody, and mouse anti-porcine CD8A-PE antibody were obtained from SouthernBiotech (SouthernBiotech, USA). Anti-mouse IgG-FITC was acquired from Sigma (Sigma, USA). Additionally, mouse monoclonal antibodies specific for the PRV gB were generously provided by Prof. Zhi-jun Tian and Jinmei Peng at our institute.

### Construction of the expression plasmids and purification of the protein

The gB and gD genes of the PRV HeN1 strain (GenBank Accession: KP098534) were codon-optimized and synthesized by Sangon Biotech (Sangon Biotech, China). Subsequently, the ectodomain of either gB or gD was cloned into the pb513B vector, which incorporates a Flag tag along with a 6× His tag. A stable CHO cell line expressing these constructs was then established as previously described (33). The CHO cells stably expressing the recombinant protein were established through puromycin selection. The recombinant protein present in the supernatant of cultured cells was collected and purified using Ni-NTA resin affinity chromatography (GenScript, USA) according to the manufacturer’s instructions. SDS-PAGE and Western blot analyses were conducted to confirm the purity of the isolated protein. The concentration of the purified proteins was quantified utilizing a BCA Protein Assay Kit (Solarbio, China), after which these proteins were stored at –80°C.

### PRV replication was inhibited by protein

To investigate the effect of gB or gD on viral replication, different concentrations of gB or gD protein were co-incubated with the PRV-EGFP reporter virus (multiplicity of infection = 0.01) on Vero E6 cells for 2 hours. After washing three times with cold PBS, the samples were cultured in DMEM containing 2% FBS. PBS was used as the control. After 36 hours of infection, the infected cells were examined under a microscope. The extent of infection was determined by quantifying the area occupied by EGFP-positive cells using Image J software.

### Design of the mouse vaccination experiments by the gB and gD protein

For the vaccination of BALB/c mice, a total of 15 female BALB/c mice were randomly assigned to three groups. The mice received intramuscular injections of 100 µL (100 µg) of purified gB or gD protein in the hind leg, or phosphate-buffered saline (PBS), at weeks 0 and 2. During the initial immunization, the proteins were emulsified with Freund’s complete adjuvant (Sigma, USA). In subsequent booster administrations, they were combined with Freund’s incomplete adjuvant (Sigma, USA). Blood samples were collected from the retro-orbital sinus using glass capillaries. Serum samples were obtained 2 weeks after booster immunization and stored at −20 °C for neutralizing antibody detection.

### Immunofluorescence assay

HEK293T cells treated with gD mRNA-LNPs or empty LNPs were washed with PBS and fixed with 4% paraformaldehyde (PFA) for 15 minutes at room temperature and washed twice with PBS as described in our previous studies (34–39). Then, the cells were permeabilized with 0.25% Triton X-100 for 10 minutes at 4°C and blocked with 2% bovine serum albumin (BSA) (Coolaber, China) for 30 minutes at 37°C. After washing three times with PBS, the cells were subsequently incubated with PRV gD-specific primary antibodies at 37°C for 1 hour and washed three times with PBS. The cells were then incubated with appropriate secondary antibodies at 37°C for 1 hour and washed three times with PBS. The cell nuclei were stained with 4′,6-diamino-2-phenylindole (DAPI) for 15 minutes at 37°C and washed three times with PBS. Finally, the fluorescence signals were detected via microscope.

### Western blot

The western blot was performed as described in our previous reports (33, 40–42). Briefly, HEK293T cell lysates or PRV gB and gD proteins were mixed with loading buffer for SDS-PAGE. The separated proteins were transferred to PVDF membranes (Millipore, USA). The membranes were blocked with 5% nonfat milk in PBS for 1 hour and incubated with an antibody overnight at 4°C, followed by washing and incubation with the appropriate secondary antibody for 1 h at room temperature. Finally, the membranes were visualized using the Odyssey CLx imaging system.

### mRNA production and LNP encapsulation

The glycoprotein D of the HeN1 strain of PRV was employed as the reference amino acid sequence (GenBank Accession: KP098534). The ectodomain (1–355 aa) was cloned into an mRNA vector, followed by *in vitro* transcription using T7 RNA polymerase and a linearized plasmid DNA template containing optimized codons for the glycoprotein D. The lipids, including cationic lipid (SM102), phospholipid with two stearoyl chains and a phosphorylcholine headgroup (DSPC), cholesterol, and DMG-2000, were dissolved in anhydrous ethanol at a mass ratio of 50:10:38.5:1.5. Subsequently, the mRNA was dissolved in an aqueous phase prepared using citrate buffer solution at pH 4.0 (50 mM). The microfluidic device (LNP-S1-L, Fluidiclab, China) was utilized to package the mRNA with an alcohol phase to water phase ratio of 1:3 (43). The resulting mixture was then diluted with RNase-free PBS buffer and concentrated through ultrafiltration employing a 30 kDa cutoff membrane. To adjust the mRNA concentration to 60 µg/mL and the sucrose concentration to 10%, an equal volume of PBS solution containing 20% sucrose was added. Finally, the gD mRNA-LNPs vaccine preparation underwent filtration through a 0.22 µm membrane before being stored at −20°C after packaging. The particle size and PDI were measured by NanoBrook 90Plus device (Brookhaven Instruments Corporation, USA) according to the instructions.

### Expression of mRNA encoding the PRV gD

HEK293T cells were seeded in 12-well plates. When the cells reached 70%–80% conﬂuence, cells were treated with 2 µg gD mRNA-LNPs. The empty LNPs were served as the control group. After 24 hours of incubation, the expression of mRNA was analyzed by IFA and western blot (30, 44).

### Design of the mouse vaccination experiments and viral challenge study

#### Safety evaluation

In all, 12 BALB/c female mice (6 weeks old) were randomly assigned to two groups of 6 mice per group. The immunization schedule is shown in Fig. 3A, one group vaccinated via intramuscular (i.m.) injection with a volume of 150 µL (10 µg) gD mRNA-LNPs at the hind leg. Mice in the control group were given 150 µL empty LNPs. A booster immunization was performed 2 weeks after primary immunization. The blood samples were obtained from the retro-orbital sinus using glass capillaries. Serums were collected at 0, 2, 3, 4, 5, 6, 7, 8, 9, and 10 weeks post-vaccination for antibody analysis. The mice were euthanized with inhaled CO_2_, and then the antigen-specific lymphocyte proliferation assays and IFN-γ detection were conducted using splenocytes that were extracted at 4 weeks post-first vaccination.

#### Intramuscular challenge

To investigate the protective efficacy of gD mRNA-LNPs against PRV challenge via intramuscular infection, 15 BALB/c female mice were randomly divided into three groups. The experimental schedule is shown in Fig. 4A, groups 1 and 2 (*n* = 5) were immunized with a volume of 150 µL gD mRNA-LNPs (10 µg) or 150 µL empty LNPs via i.m. injection at the hind leg, respectively. Group 3 was given a volume of 150 µL empty LNPs and designated as the negative control. All groups were immunized twice with the same dose, 14 days apart. The blood samples were obtained from the retro-orbital sinus using glass capillaries. Serums were collected for antibody analysis. Groups 1 and 2 were challenged with a volume of 100 µL PRV HeN1 strain (2 × 10^4^ PFU) via i.m. injection at the hind leg at 28 days post the first immunization. Group 3 served as the mock challenge group and was not subjected to viral challenge during experiments. After the challenge, the mice were observed daily, and the clinical symptoms were scored as in previous research (22). At 14 days post-challenge (dpc), the surviving mice were euthanized with inhaled CO_2_, and samples of six tissues, including brain, lung, heart, liver, spleen, and kidney, were collected for viral detection via quantitative real-time polymerase chain reaction (qRT-PCR). The brain and lung were subjected to histopathological examination following hematoxylin and eosin (H&E) staining.

#### Mucosal challenge

To investigate the mucosal protective effects of gD mRNA-LNPs against PRV challenge via the respiratory route, 10 male and 5 female BALB/c mice were randomly assigned to three groups. The experimental schedule is illustrated in Fig. 5A. Groups 1 and 2 (*n* = 5) received immunizations with gD mRNA-LNPs or empty LNPs, respectively, while Group 3 was administered empty LNPs and served as the negative control. The immunization dose, route of administration, and intervals for the mice were conducted as previously described. At 28 days post-initial immunization, Groups 1 and 2 underwent intranasal challenge with a volume of 20 µL containing the PRV HeN1 strain (1 × 10^3^ PFU). Following the challenge, daily observations were made on the mice to assess clinical symptoms, which were subsequently scored. 14 days post-challenge (dpc), surviving mice were euthanized using inhaled CO_2_; tissues were then collected for viral detection through qRT-PCR analysis.The brain and lung were subjected to histopathological examination following hematoxylin and eosin (H&E) staining.

### Design of the pig vaccination experiments and viral challenge study

#### Safety evaluation

In all, 15 piglets (28 days old, 9 males and 6 females) were free of PRV, PRRSV, ASFV, PCV2, PCV3, and CSFV. The piglets were randomly divided into three groups (*n* = 5). The experimental schedule is shown in Fig. 6A, group 1 was immunized via intramuscular (i.m.) injection with a volume of 1 mL (50 µg) gD mRNA-LNPs at neck muscles. Groups 2 and 3 were given a volume of 1  mL empty LNPs at the neck muscles. The vaccinated piglets also received a booster dose at 14 dpv. The piglets’ body weights were measured weekly. Furthermore, serum samples were collected from each piglet at 0, 7, 14, 21, and 28 dpv for antibody detection. The piglet peripheral blood lymphocytes were isolated to measure the antigen-specific T-cell proliferation and IFN-γ detection.

#### Intramuscular challenge

At 28 dpv, groups 1 and 2 were challenged with a volume of 1 mL PRV HeN1 strain via intramuscular injection (1.0 × 10^6.0^ PFU per piglet) in the neck muscles. Group 3 served as the mock challenge group and was not subjected to viral challenge during experiments. The animals were recorded for clinical signs and rectal temperatures. The clinical symptoms were scored as in previous research (45). The rectal temperature ≥40.5°C was defined as fever. Furthermore, the blood samples, nasal swab, and anal swab were collected from piglets at 0, 3, 5, 7, 10, and 14 days post-challenge (dpc). All surviving piglets were euthanized using Zoletil 50 (Virbac, France) (7 mg/kg) via i.m. injection at the neck muscles and autopsied at 14 dpc. The serum samples were collected from individual piglets to test for the presence of viremia. The nasal swab, anal swab, and samples of nine tissues, including brain, heart, liver, spleen, lung, kidney, tonsil, submandibular lymph node, and inguinal lymph node, were collected for viral detection via qRT-PCR. The brain and lung were collected and subjected to histopathology and immunohistochemistry analysis. Our institute is equipped with a team of professional pathologists. When we provide tissue samples, we assign random numbers and do not disclose detailed information to ensure the integrity of the analysis. Consequently, histopathological slides are examined in a blinded manner.

#### Enzyme-linked immunosorbent assay

PRV gD-, gB-, and gE-specific antibodies were detected via indirect enzyme-linked immunosorbent assay (ELISA) as shown in previous research (46–48). The proteins were expressed in CHO cells, and purified proteins (100 ng/well) were coated onto 96-well ELISA plates (Corning, USA) overnight at 4°C. The plates were blocked with 3% BSA for 2 h at 37°C. The diluted serum sample (1:200) was added to the plates and incubated at 37°C for 1 h. After washing three times with PBST, 5 min for each time, and incubating at 37°C for 45 min with HRP-conjugated secondary antibodies (ZSGB-BIO, China). After another washing step, TMB solution (Abcam, USA) was added to each well and incubated under dark conditions for 10 min at room temperature. The reaction was stopped with 2 M H_2_SO_4_, and the absorbance was read on a microtiter plate reader (PE, USA) at 450 nm within 10 min.

### Virus-neutralizing antibody detection

Serum-neutralizing antibodies against PRV were determined by performing a neutralization assay. The assay was performed as previously described with minor modifications (30, 49). Serum samples from mice and piglets were inactivated by heating at 56°C for 30 min and then twofold serial dilutions with DMEM. A similar amount of PRV HeN1 EGFP strain, HeN1 strain, SC strain, TJ strain, or Bartha K61 strain (200 TCID_50_) was mixed thoroughly with 50 µL of the diluted inactivated serum for 2 h at 37°C. Subsequently, the mixtures of 100 µL were added to a 96-well plate with monolayer Vero E6 cells for 2 h at 37°C. Two hours later, the cells were washed three times with cold PBS and cultured in DMEM containing 2% FBS. Blank cells were set up as a negative control. The plates were incubated for 48 hours at 37°C with 5% CO_2_. Neutralizing antibodies were calculated using the Reed-Muench method.

### T-lymphocyte proliferation assay

The splenic lymphocytes from mice or peripheral blood lymphocytes from piglets at 28 dpv were isolated to assess the proliferation of PRV gD-specific T cells using the CellTrace Violet Cell Proliferation Kit (Invitrogen, USA), following the manufacturer’s instructions. The splenic lymphocytes or peripheral blood lymphocytes were stained with CellTrace Violet. When they are stimulated by specific antigens, they undergo division and proliferation, leading to a decrease in the violet fluorescence signal. This reduction can be utilized to assess cell proliferation status. Subsequently, the stained cells by CellTrace Violet were seeded into a 12-well round-bottom plate at a density of 1,000,000 cells per well and stimulated with gD mRNA-LNPs (10 µg), gE mRNA-LNPs (10 µg), or empty LNPs at 37°C with 5% CO_2_ for 72 hours. Empty LNPs and gE mRNA-LNPs stimulated cells were used as negative controls. The splenic lymphocytes from mice were then stained with PE/Cyanine5 anti-mouse CD3 antibody (Biolegend, USA), APC/Cyanine7 anti-mouse CD8a antibody (Biolegend, USA), and FITC rat anti-mouse CD4 antibody (BD-Pharmingen, USA). The peripheral blood lymphocytes from piglets were stained with mouse anti-porcine CD3E-SPRD antibody (SouthernBiotech, USA), mouse anti-porcine CD4-FITC antibody (SouthernBiotech, USA), and mouse anti-porcine CD8A-PE antibody (SouthernBiotech, USA). After three washes with PBS, flow cytometric analysis was performed using an Apogee flow cytometer (UK). Data analysis was conducted using FlowJo version 10.8.

### Enzyme-linked immunospot assay

The cellular immune responses in the vaccinated piglets were evaluated using IFN-γ precoated enzyme-linked immunospot (ELISPOT) kits (Mabtech, USA) following the manufacturer’s instructions. Briefly, the plates were blocked with RPMI 1640 medium (Gibco, USA) containing 10% FBS and incubated for 30 minutes. Peripheral blood lymphocytes from piglets were seeded at a density of 300,000 cells per well and stimulated with gD mRNA-LNPs (5 µg) or empty LNPs followed by incubation at 37°C with 5% CO_2_ for 48 hours.

Empty LNPs stimulated cells were used as negative controls. The plates were washed five times with PBS and then incubated at room temperature for 2 hours with a detection antibody. Streptavidin-HRP was added to the plates and incubated for an additional hour. After final washes, the TMB substrate solution was applied and subsequently stopped with water. The air-dried plates were analyzed using an ELISpot reader (AID, Germany) to determine the number of spot-forming cells per million cells.

### IFN-γ detection

The spleen lymph single-cell suspensions from mice were seeded into 12-well plates at a density of 1,000,000 cells per well and incubated with gD mRNA-LNPs (10 µg) and Empty LNPs for 72 hours at 37°C. The cells stimulated by empty LNPs were used as the negative control. Subsequently, the cells were centrifuged and the supernatants were collected to measure IFN-γ levels using commercial mouse IFN-γ ELISA kits (Cusabio, China), following the manufacturer’s instructions. The concentration of IFN-γ in the samples was determined based on standard curves.

### Quantitative real-time polymerase chain reaction

The viral DNA from blood, tissues, and swab samples was extracted using the TIANamp Genomic DNA Kit (Tiangen, China) following the manufacturer’s instructions. Quantification of the copy numbers of PRV’s gB gene was performed using qRT-PCR, as previously described in our reports (23, 29, 40).

### Assessment of the broad-spectrum efficacy of gD mRNA vaccine against various PRV strains

A total of 37 gD amino acid sequences were obtained from the NCBI GenBank database, comprising 4 sequences of genotype I, 5 sequences of genotype II classic strains, and 28 sequences of genotype II variant strains. Subsequently, the MegaAlign software was employed to analyze the conservation of the gD protein amino acid sequence across various PRV strains, while phylogenetic analysis was conducted using MEGA software. Sera collected at 28 days post-vaccination (dpv) from immunized mice and piglets were evaluated through IFA and neutralization assays against several PRV strains, including HeN1, SC, TJ, and Bartha K61, which represent different branches in the phylogenetic tree.

### Statistical analysis

Statistical analysis was conducted via GraphPad Prism 8.0 software. Statistical significance was analyzed via t tests. A *P* value < 0.05 was considered statistically significant. Statistical significance is indicated by * *P* < 0.05, ** *P* < 0.01, *** *P* < 0.001, and **** *P* < 0.0001.

## Data Availability

All data pertinent to this work are contained within this article.

## SUPPLEMENTAL MATERIAL

Supplemental figures jvi.01472-25-s0001.docx Fig. S1 to S6 and sequence.

ASM does not own the copyrights to Supplemental Material that may be linked to, or accessed through, an article. The authors have granted ASM a non-exclusive, world-wide license to publish the Supplemental Material files. Please contact the corresponding author directly for reuse.
