# Supplementary material for: A glycoprotein D-targeted lipid nanoparticle-encapsulated mRNA vaccine elicits strong protective immunity against pseudorabies virus
Source: J Virol. 2025 Nov 6;99(11):e01472-25. doi: 10.1128/jvi.01472-25 (PMC12645995; doi:10.1128/jvi.01472-25)
Supplement: Supplemental figures — Fig. S1 to S6 and sequence. [file jvi.01472-25-s0001.docx]

**Supplementary data**


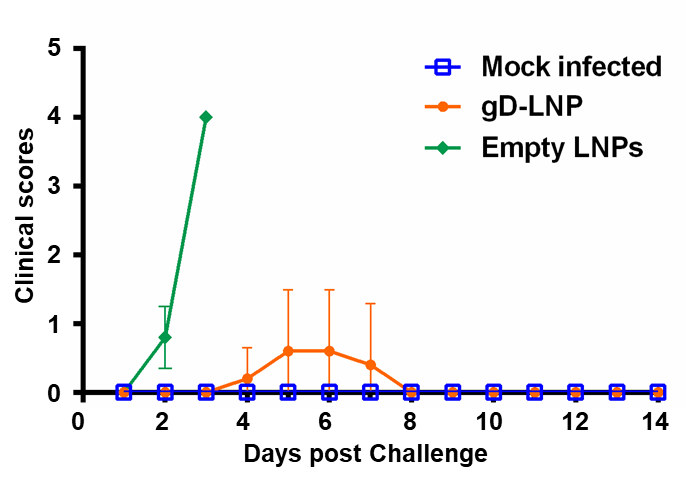


**Figure S1.** The clinical score of mice immunized with mRNA-LNPs or empty LNPs after challenge.


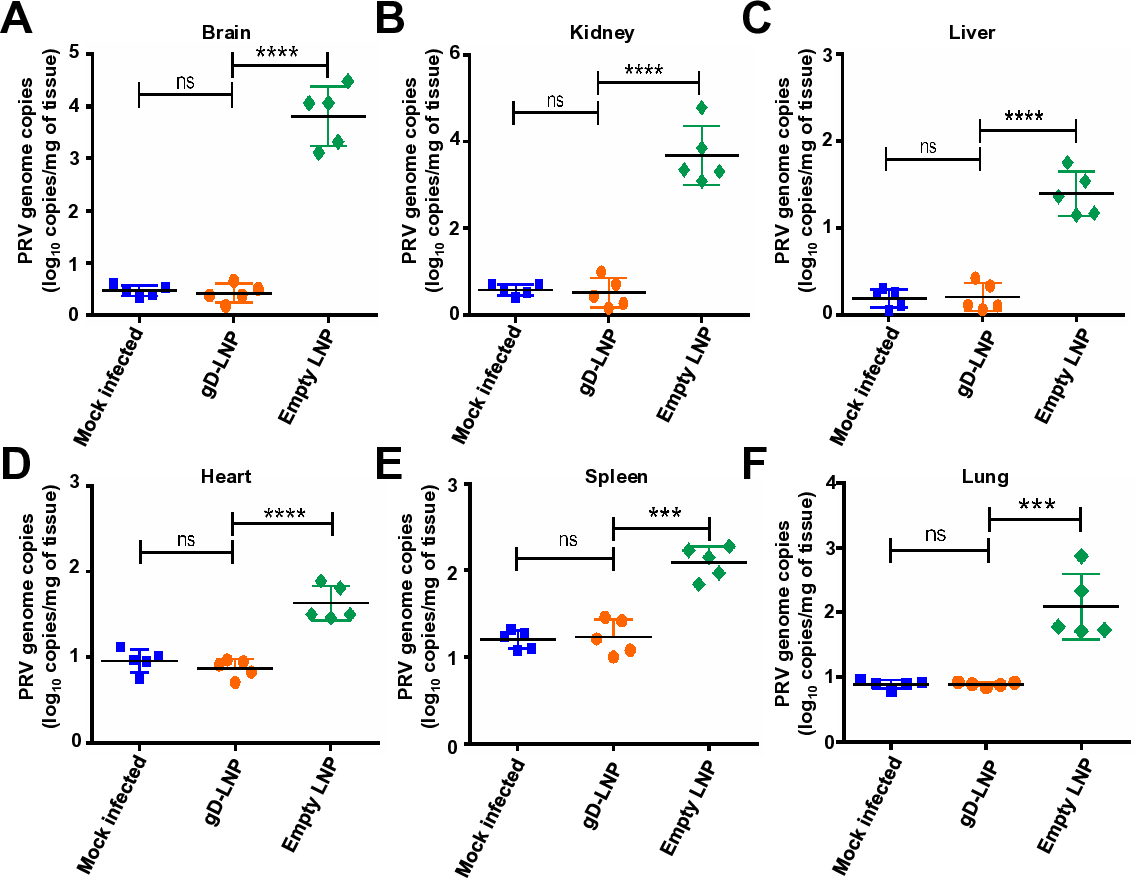


**Figure S2.** The viral loads quantified by real-time PCR in indicated mice tissues, including (**A**) brain, (**B**) kidney, (**C**) liver, (**D**) heart, (**E**) spleen, and (**F**) lung. The data represent the means ± standard deviation (SD) from five animals.


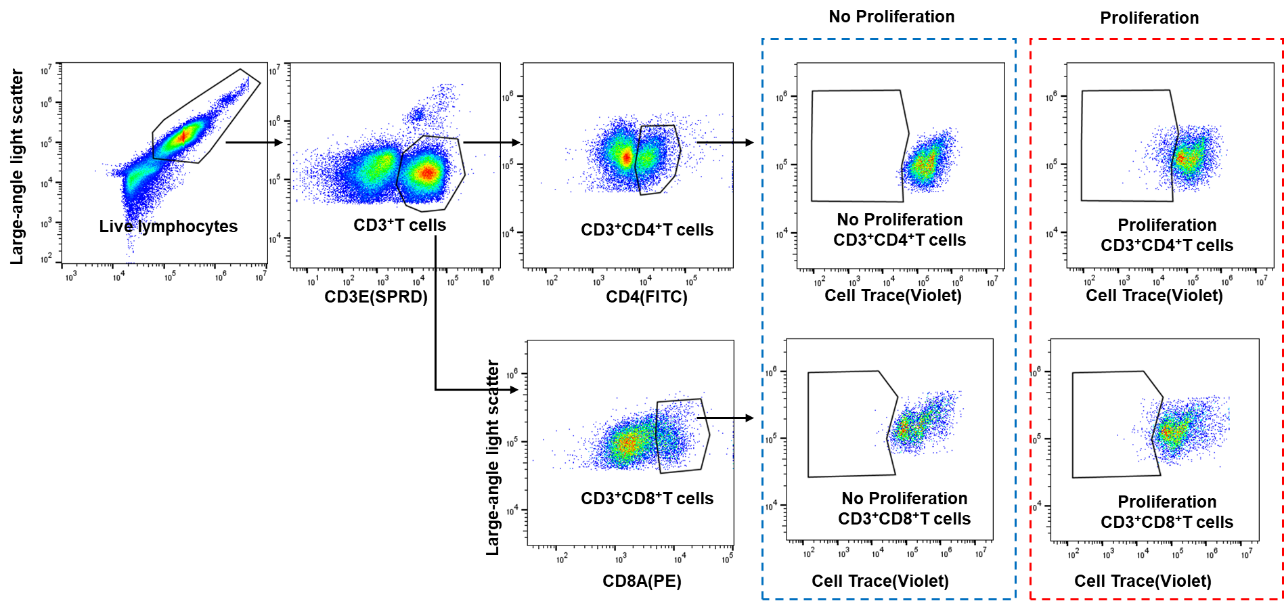


**Figure S3.** **Detection of** **CD3^+^CD4^+^ T cells, or CD3^+^CD8^+^ T cells response.** Peripheral blood lymphocytes were isolated from piglets and stained using the CellTrace™ Violet Cell Proliferation Kit. Subsequently, 1,000,000 stained cells were stimulated with gD-LNP (10 μg) at 37°C in a 5% CO2 atmosphere for 72 hours. Concurrently, gE-LNP (10 μg) and empty LNPs were utilized as negative controls for stimulation. Following this incubation period, the cells were stained with anti-porcine antibodies targeting CD3,CD4,and CD8, in order to assess the gD-specific CD4^+^ T cell response and the CD8^+^ T cell response. After three washes with PBS, flow cytometric analysis was conducted to assess the results.


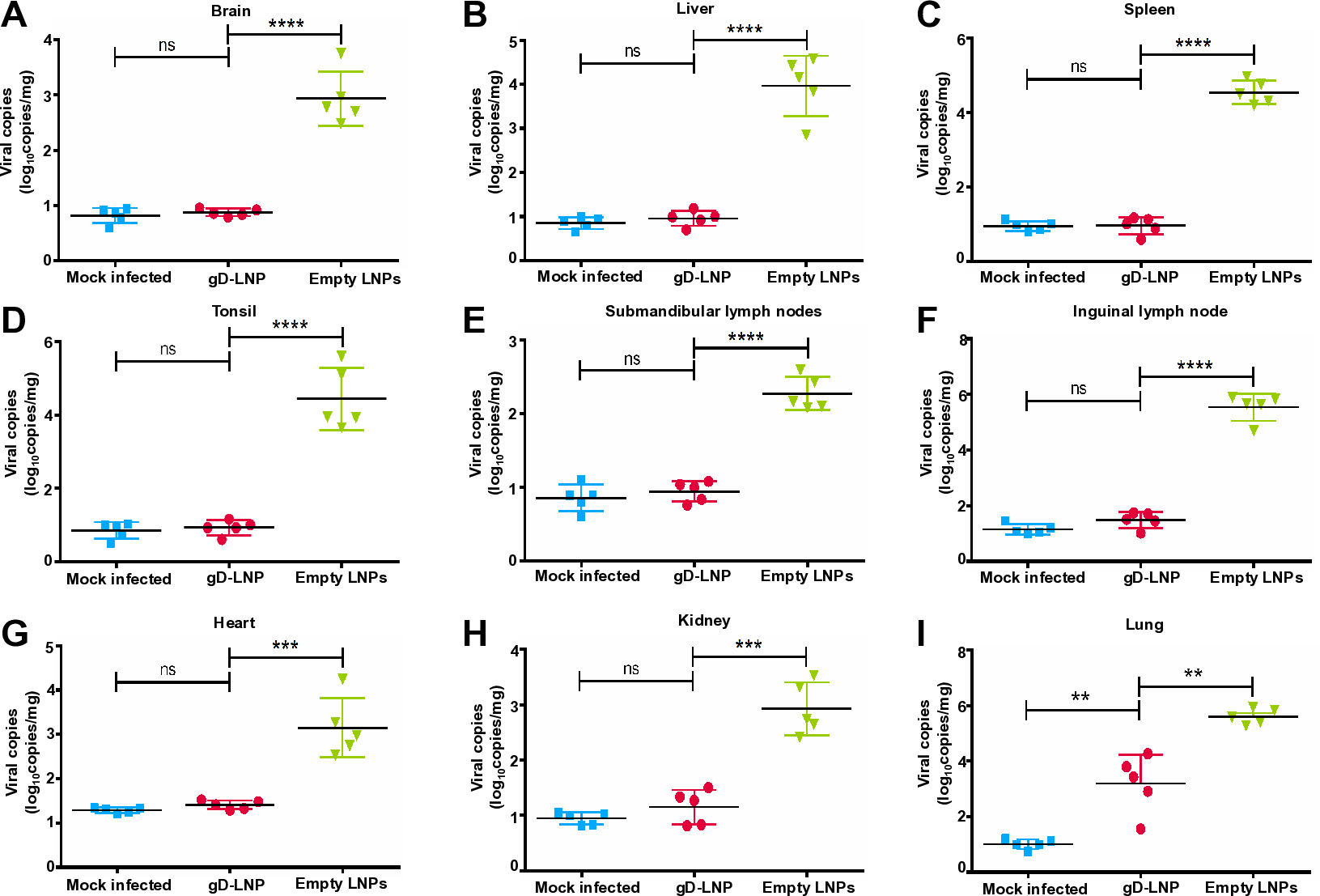


**Figure S4.** The viral loads quantified by real-time PCR in indicated piglet tissues, including (**A**) brain, (**B**) liver, (**C**) spleen, (**D**) tonsil, (**E**) submandibular lymph nodes, (**F**) inguinal lymph nodes, (**G**) heart, (**H**) kidney, and (**I**) lung. The data represent the means ± standard deviation (SD) from five animals.


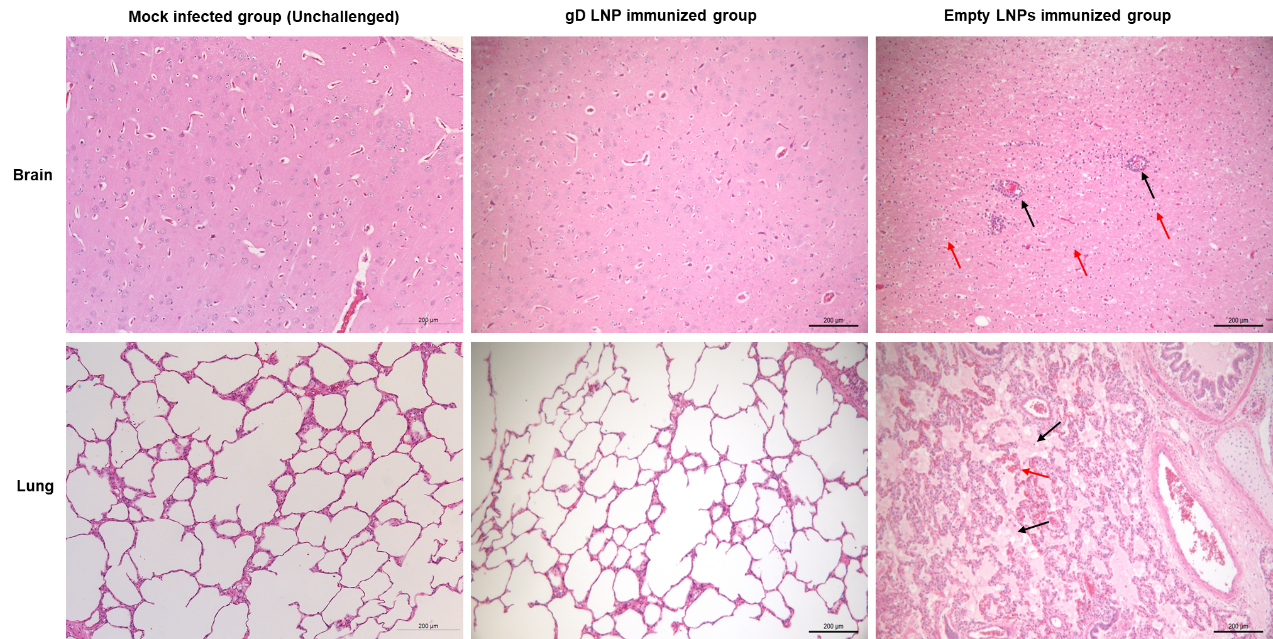


**Figure S5. Hematoxylin and Eosin (H&E) staining of brain and lung tissues.** Microscopic lesions in the brain and lung of different groups following PRV challenge are presented. Histopathological alterations induced by the PRV HeN1 strain are indicated by arrows. Scale bars, 200 μm. The LNPs group of pig brain cross-sections demonstrates extensive and diffuse infiltration of glial cells in both the gray matter and white matter (red arrow), accompanied by perivascular inflammatory cell infiltration (black arrow). In the LNPs group of pig lung, there is evidence of capillary congestion within the alveolar walls (red arrow) as well as an accumulation of serous fibrinous exudate in the alveolar cavities (black arrow). This experiment was conducted three times, and a representative result is presented.


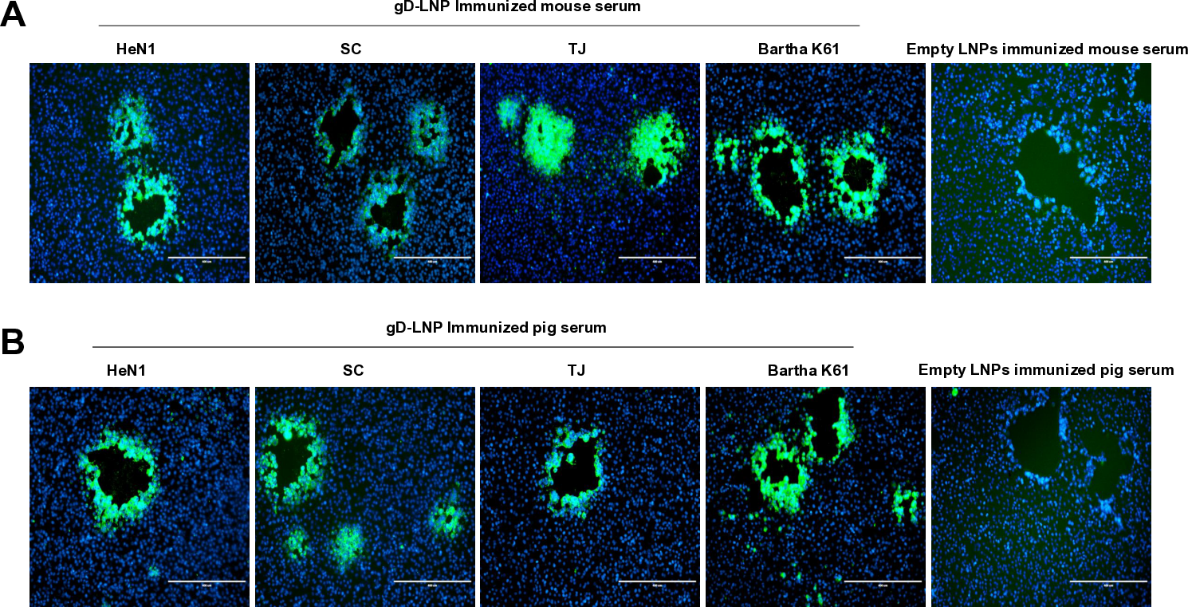


**Figure S6.** The indirect immunofluorescence assays (IFA) with sera collected at 28 dpv from gD mRNA-vaccinated mice (**A**) and piglets (**B**). The empty LNPs immunized serum were used as negative controls. Vero E6 cells were infected with HeN1 strain, SC strain, TJ strain, Bartha K61 strain and fixed in paraformaldehyde prior to IFA. Scale bars, 400 μm. This experiment was conducted three times, and a representative result is presented.

**Supplementary sequence**

taatacgactcactataaggaaataagagagaaaagaagagtaagaagaaatataagagccaccgctagcctcgaggccaccatggacgccatgaagagggggctgtgctgcgtgctgctgctgtgcggagccgtgttcgtgagcgcctccATGCTGCTGGCCGCCCTGCTGGCCGCTCTGGTGGCCCGCACCACCCTGGGCGCCGACGTGGACGCCGTGCCCGCCCCCACCTTCCCCCCCCCCGCCTACCCCTACACCGAGAGCTGGCAGCTGACCCTGACCACCGTGCCCAGCCCCTTCGTGGGGCCCGCCGACGTGTACCACACGCGCCCCCTGGAGGACCCCTGCGGCGTGGTGGCCCTGATCAGCGACCCACAGGTGGACAGGCTGCTGAATGAGGCCGTGGCCCACCGCCGGCCCACCTACCGCGCCCACGTGGCCTGGTAtCGGATCGCCGACGGCTGCGCCCACCTGCTGTACTTCATCGAGTATGCCGACTGTGACCCCCGGCAGATCTTCGGCCGCTGCCGGCGCCGGACCACCCCCATGTGGTGGACCCCCAGCGCCGACTACATGTTCCCCACCGAGGACGAGCTGGGCCTCCTGATGGTGGCCCCCGGCCGGTTCAACGAAGGCCAGTACCGGAGGCTGGTGTCCGTGGACGGGGTGAACATCCTGACCGACTTCATGGTGGCCCTGCCCGAGGGCCAGGAGTGCCCCTTCGCCCGCGTTGATCAGCATAGAACTTATAAATTTGGCGCTTGTTGGTCCGACGACTCCTTCAAGAGGGGTGTGGACGTGATGCGGTTCCTGACCCCCTTCTACCAGCAGCCCCCCCACCGCGAGGTGGTGAACTACTGGTAtCGCAAGAACGGCAGGACCCTGCCCCGCGCCTACGCCGCCGCCACCCCCTACGCCATCGACCCCGCCCGGCCTTCCGCCGGGAGCCCTCGCCCCCGCCCCCGGCCCCGCCCCCGCCCCCGCCCAAAGCCCGAGCCCGCTCCCGCCACCCCCGCCCCCCCCGGCCGGCTGCCCGAGCCCGCCACCCGcGACCACGCCGCCGGCGGCCGCCCCACCCCCCGCCCCCCCCGCCCCGAaACCCCCCACCGGCCCTTCGCCCCCCCCGCCGTGGTGCCCAGCGGGTGGCCCCAGCCCGCCGAGCCCTTCCCCCCTAGAACTACCGCCGCCCCAGGAGTGAGTAGACATAGATCTGTGtgaggtaccgatatctgataataggctggagcctcggtggccatgcttcttgccccttgggcctccccccagcccctcctccccttcctgcacccgtacccccgtggtctttgaataaagtctgaaaaaaaaaaaaaaaaaaaaaaaaaaaaaaaaaaaaaaaaaaaaaaaaaaaaaaaaaaaaaaaaaaaaaaaaaaaaaaaaaaaaaaaaaaaaaaaaaaaaaaaaa

T7 promoter

5’UTR

Kozak sequence

tPA

PRV gD

3’UTR

Poly A
